# Cerebellum Involvement in Visuo-vestibular Interaction for the Perception of Gravitational Direction: A Repetitive Transcranial Magnetic Stimulation Study

**DOI:** 10.1523/ENEURO.0111-25.2025

**Published:** 2025-07-30

**Authors:** Keisuke Tani, Hiroaki Tanaka, Akimasa Hirata, Yosuke Nagata, Nobuhiko Mori, Koichi Hosomi, Akiyoshi Matsugi

**Affiliations:** ^1^Faculty of Psychology, Otemon Gakuin University, Ibaraki, Osaka 567-8502, Japan; ^2^Graduate School of Human and Environment Studies, Kyoto University, Kyoto, 606-8501, Japan; ^3^Department of Physical Medicine and Rehabilitation, Kansai Medical University Hospital, Hirakata City, Osaka 573-1191, Japan; ^4^Department of Physical Medicine and Rehabilitation, Kansai Medical University, Hirakata City, Osaka 573-1010, Japan; ^5^Center of Biomedical Physics and Information Technology, Nagoya Institute of Technology, Nagoya 466-8555, Japan; ^6^Department of Neurosurgery, Osaka University Graduate School of Medicine, Suita City, Osaka 565-0871, Japan; ^7^Faculty of Rehabilitation, Shijonawate Gakuen University, Daitou City, Osaka 574-0011, Japan

**Keywords:** cerebellum, gravity, transcranial magnetic stimulation, visual vertical, visuo-vestibular integration

## Abstract

Accurate perception of the direction of gravity relies on the integration of multisensory information, particularly from the visual and vestibular systems, within the brain. Although a recent study of patients with cerebellar degeneration suggested a cerebellar role in visuo-vestibular interaction in the perception of gravitational direction, direct evidence remains limited. To address this gap, we conducted two experiments with 42 healthy participants to evaluate the impact of repetitive 1 Hz transcranial magnetic stimulation (rTMS) over the posterior cerebellar vermis on visual dependency, quantified by the subjective visual vertical bias induced by rotating optokinetic stimulation (OKS). Electric field simulations in high-resolution generic head models were used to ensure focal stimulation of the cerebellum at the group level. The results demonstrated that the rTMS applied to the vermis significantly attenuated the OKS-induced shift in visual vertical (SVV) bias. This effect was not observed when stimulation was applied to the early visual cortex (V1–2) or the cerebellar hemisphere. Also, the vermis rTMS had no effect on the judgment precision in the absence of visual motion cues, suggesting that the rTMS may reduce visual weight in visuo-vestibular processing by increasing visual motion noise rather than affecting vestibular function. These findings suggest a direct involvement of the cerebellar vermis in the visuo-vestibular interaction underlying the perception of gravitational direction, providing new insights into cerebellar contributions in human spatial orientation.

## Significance Statement

The cerebellum has been implicated in multisensory integration for spatial orientation, but its direct role in visuo-vestibular interactions remains limited. Using 1 Hz rTMS, we demonstrated that stimulation of the cerebellar vermis significantly reduced visual dependency in the perception of gravitational direction, as measured by the subjective visual vertical bias induced by rotating optokinetic stimulation. This effect was absent when adjacent areas, such as the early visual cortex and cerebellar hemisphere, were stimulated. These results suggest that the cerebellar vermis is directly involved in visuo-vestibular interactions, providing new insights into the cerebellar contribution to spatial orientation in humans.

## Introduction

Awareness of the direction of gravity provides a fundamental basis for the perception of external events and motor control on Earth. A large body of psychophysical evidence has shown that the brain integrates different types of sensory information, especially vestibular and visual information, to internally estimate the direction of gravity, likely in a statistically optimal manner (Angelaki et al., 2009; Niehof et al., 2019; see also Kheradmand and Winnick, 2017 for a review). However, the neural substrates underlying visuo-vestibular interaction in the context of the perception of gravitational direction are largely unclear.

The otolith organs in the inner ear, comprising the utricle and saccule, respond to linear acceleration and constitute an essential sensor for detecting gravitational direction with respect to the head (Goldberg et al., 2012). However, because gravitational (tilt) and translational accelerations are physically indistinguishable (Einstein, 1908), the otolith signal provides ambiguous information regarding the gravitational direction. To resolve this (tilt–translation) ambiguity, the brain seems to track the direction of head tilt relative to gravity on the basis of angular velocity signals from the visual system, as well as the semicircular canal, and integrate this information with otolith signals (Laurens and Angelaki, 2011; Dakin et al., 2018; MacNeilage and Glasauer, 2018; Diaz-Artiles and Karmali, 2021). In fact, it is known that large-field angular visual motion (i.e., rotating optic flow) leads to a strong bias in the perceived direction of gravity toward its direction (Dichgans et al., 1972).

Recently, Dakin et al. (2018) showed that the bias of the perceived direction of gravity induced by large-field visual motion was much greater (interpreted as “increased visual dependency”) in patients with spinocerebellar ataxia type 6 (SCA6) and slowly progressive degeneration limited to the cerebellum than in age-matched healthy participants. This finding suggests that the cerebellum subserves the visuo-vestibular interaction for the perception of gravitational direction. However, recent studies have reported reduced functional activity not only in the cerebellum but also in various cortical regions (Kang et al., 2017), as well as changes in functional/effective connectivity between the cerebellum and cortical regions as symptoms progress (Falcon et al., 2015; Kang et al., 2017). These findings suggest that the increased visual dependency observed in SCA6 patients may be attributed to compensatory functional changes in cerebral cortical areas, such as the visual cortex, following cerebellar degeneration. Therefore, whether the cerebellum is directly involved in visuo-vestibular interaction remains unclear.

To address this issue, we performed two experiments in healthy participants using low-frequency (1 Hz) repetitive transcranial magnetic stimulation (rTMS), which can induce focal and short-term modulation of neural activity in targeted brain regions (Pascual-Leone, 1999; Kvamme et al., 2022). Neurophysiological studies have demonstrated that cerebellar brain inhibition, which reflects the inhibitory mechanism of the cerebellum on the motor cortex, is suppressed (i.e., de-inhibited) by 1 Hz rTMS over the cerebellum (Oliveri et al., 2005; Fierro et al., 2007; Popa et al., 2010). Additionally, 1 Hz cerebellar rTMS has been shown to disrupt motor learning (Lien et al., 2022; Matsugi et al., 2023) and reduce perceptual and cognitive performance (Fierro et al., 2007; Schutter and van Honk, 2009). Therefore, 1 Hz rTMS over the cerebellum generally suppresses cerebellar function (Gatti et al., 2023).

Therefore, we hypothesized that if the cerebellum contributes to visuo-vestibular interaction, then increased visual dependency would be observed after 1 Hz cerebellar rTMS, as seen in patients with SCA6. We quantified visual dependency when estimating the gravitational vertical before and after rTMS exposure over the posterior cerebellar vermis, which has been shown to be involved in both vestibular (Stephan et al., 2005) and visual motion processing (Cattaneo et al., 2014) and evaluated the effect of rTMS on visual dependency. To confirm the specific involvement of the vermis in the visuo-vestibular interaction, we further examined the effect of rTMS over adjacent cortical areas, namely, the early visual cortices (V1–2, Experiment 1) and the cerebellar hemisphere (Experiment 2).

## Materials and Methods

### Participants

In total, 22 (mean ± standard deviation age: 21.3 ± 4.3 years; 10 women, 4 left-handers) and 20 (mean age, 19.8 ± 0.9 years; 3 women, 10 left-handers) healthy volunteers participated in Experiments 1 and 2, respectively. Five participants were included in both the experiments. Handedness was determined using the Edinburgh Handedness Inventory (Oldfield, 1971). All participants reported normal vision and no neurological, visual, vestibular, or motor disorders (current or historic). The study protocol was approved by the Ethics Committee. Prior to the experiment, all participants provided written informed consent, in accordance with the Declaration of Helsinki.

### General experimental design

In this study, the visual dependency in the perception of gravitational direction was quantified by analyzing the effects of rotating optokinetic stimulation (OKS) on performance in a subjective visual vertical (SVV) judgment task (see below) and the effects of rTMS to specific brain regions on task performance.

Experiments 1 and 2 consisted of three and two sessions, respectively. In each experimental session, 1 Hz rTMS was applied to the cerebellar vermis (vermis condition), early visual cortex (V1–2 condition), or air (sham condition) in Experiment 1, whereas it was applied to the right cerebellar hemisphere (hemisphere condition) or to the air (sham condition) in Experiment 2. These experimental sessions were conducted on different days, with intervals of at least 3 d to avoid carry-over effects of prior rTMS. The order of the rTMS conditions was randomized across the participants.

**Figure 1.**
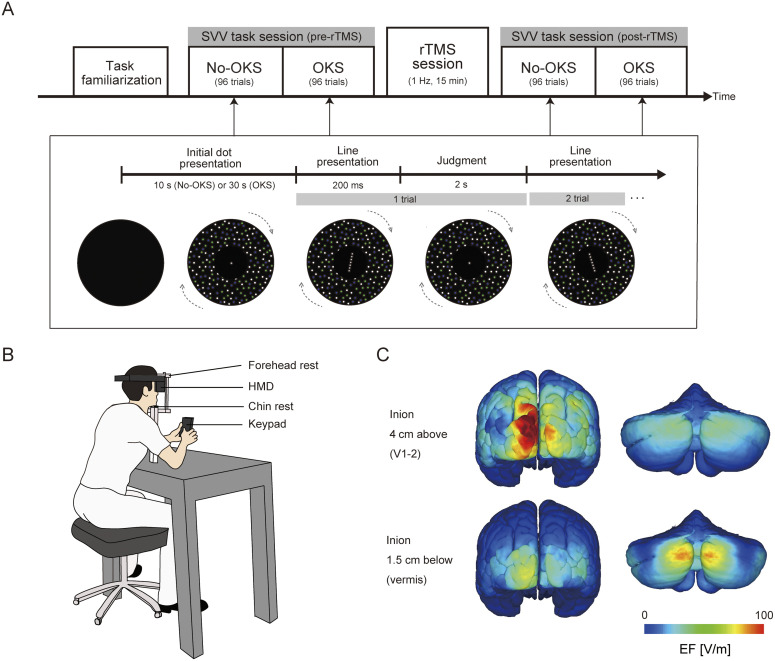
***A***, Task procedure on each experimental day. In each SVV task session before or after the rTMS session, the participants judged whether a presented line of dots was tilted in the CW or CCW direction with respect to the gravitational vertical, whereas visual background dots were either rotated (OKS condition for CW group shown as an example; denoted by the gray dashed arrows) or not rotated (No-OKS condition). They performed 96 trials for each SVV task session. One of the three rTMS conditions (vermis, V1–2, sham) was assigned to the rTMS session. ***B***, Experimental setup. Participants wore the HMD goggle and held the keypad to indicate line orientation during the SVV task session. ***C***, The estimated EF distribution (V/m) for each coil placement assigned to the V1–2 or vermis condition (see Extended Data Figs. 1-1, 1-2 shows sagittal slices of the EF distribution in the vermis and V1–2 conditions. Extended Data Figure 1-5 represents the depth-dependent changes in EF from the coil surface in the vermis condition. See Extended Data Figures 1-3 and 1-6 for the EF distribution over the ventral (caudal) region of the cerebellum in the vermis condition in Experiment 1 and the EF distribution in the hemisphere condition (1 cm below and 3 cm to the right of the inion) in Experiment 2, respectively.) EF, electric field; SVV, subjective visual vertical; OKS, optokinetic stimulation; rTMS, repetitive transcranial magnetic stimulation; CW, counter-clockwise; CCW, counter-clockwise; HMD, head-mounted display; ROI, region of interest.

On each experimental day, participants performed the SVV judgment task for 96 trials in the no-OKS condition with a stationary visual background (annulus of dots), followed by 96 trials in the OKS condition with the visual background rotating in the frontal plane (Fig. 1*A*, top panel, SVV task session). This combination was performed before and after rTMS exposure for 15 min, i.e., 96 trials × 2 OKS conditions × 2 phases = 384 trials each day (Fig. 1*A*, top panel). During the rTMS session, the participants were asked to relax in a prone position on the bed. To familiarize themselves with the experimental task, participants performed 14 trials of the SVV judgment task in each condition (No-OKS and OKS conditions), prior to the pre-rTMS phase, on each day (Fig. 1*A*, top panel, task familiarization).

10.1523/ENEURO.0111-25.2025.f1-1 Figure 1-1 Download Figure 1-1, DOCX file.

**Figure 2.**
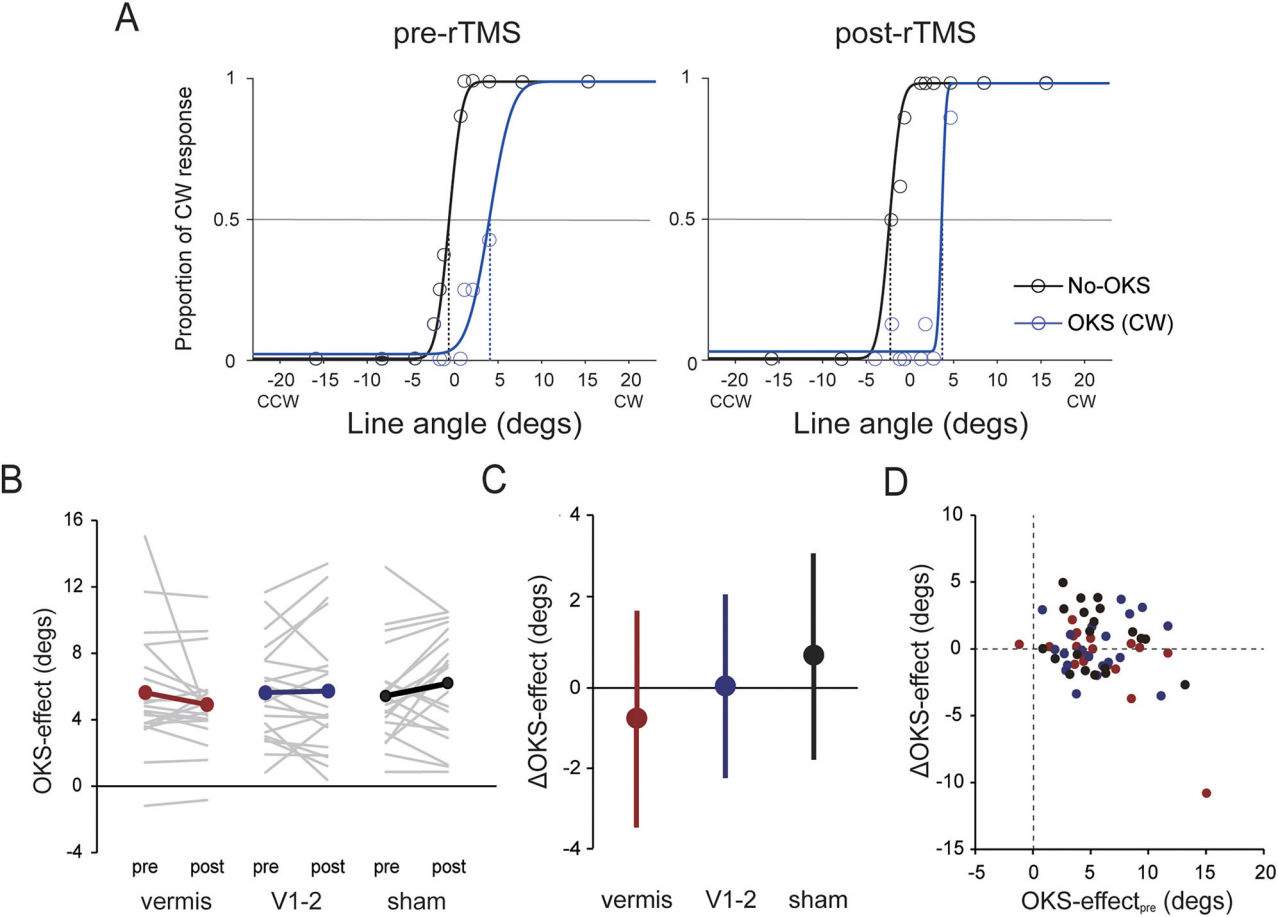
***A***, SVV task performance in each OKS condition of the sham condition for a representative participant (Sub ID = 3, CW group). Each color (black, No-OKS condition; blue, OKS condition) of circles and lines represent the proportion of CW responses for each line angle and fitted psychometric function, respectively. The intersection of each psychometric function with the horizontal gray line (response proportion = 0.5) indicates the SVV angle, as represented by the bottom of each dashed line. ***B***, The OKS-effect on SVV for each phase and rTMS condition. Gray thin lines and colored (red, vermis; blue, V1–2; black, sham) thick lines represent individual and group mean values, respectively. ***C***, ΔOKS-effect for each rTMS condition. Error bars represent the standard deviation. ***D***, Relationship between the OKS-effect in the pre-rTMS phase (OKS-effect_pre_) and ΔOKS-effect. Each plot shows the individual-level data for each rTMS condition (red, vermis; blue, V1–2, black, sham). SVV, subjective visual vertical; OKS, optokinetic stimulation; rTMS, repetitive transcranial magnetic stimulation; CW, clockwise.

10.1523/ENEURO.0111-25.2025.f1-2 Figure 1-2 To check the focality of stimulation for each coil placement (1.5 cm below and 4 cm above the inion for the Vermis and V1-–2 conditions, respectively), the highest EF among the points (*peak EF*) was evaluated and then the ratio of the EF at each point to the peak EF for the max and mean EFs and for each ROI was calculated (Table 2). The EF ratio was high (above 0.9) for the target ROIs (shown in bold), whereas it was relatively low (below 0.8) for the non-target ROIs (shown in italics) for both conditions. Ratio of EF for the target and non-target ROIs were represented in bold and italics, respectively. *: the point with the peak EF. Download Figure 1-2, DOCX file.

**Figure 3.**
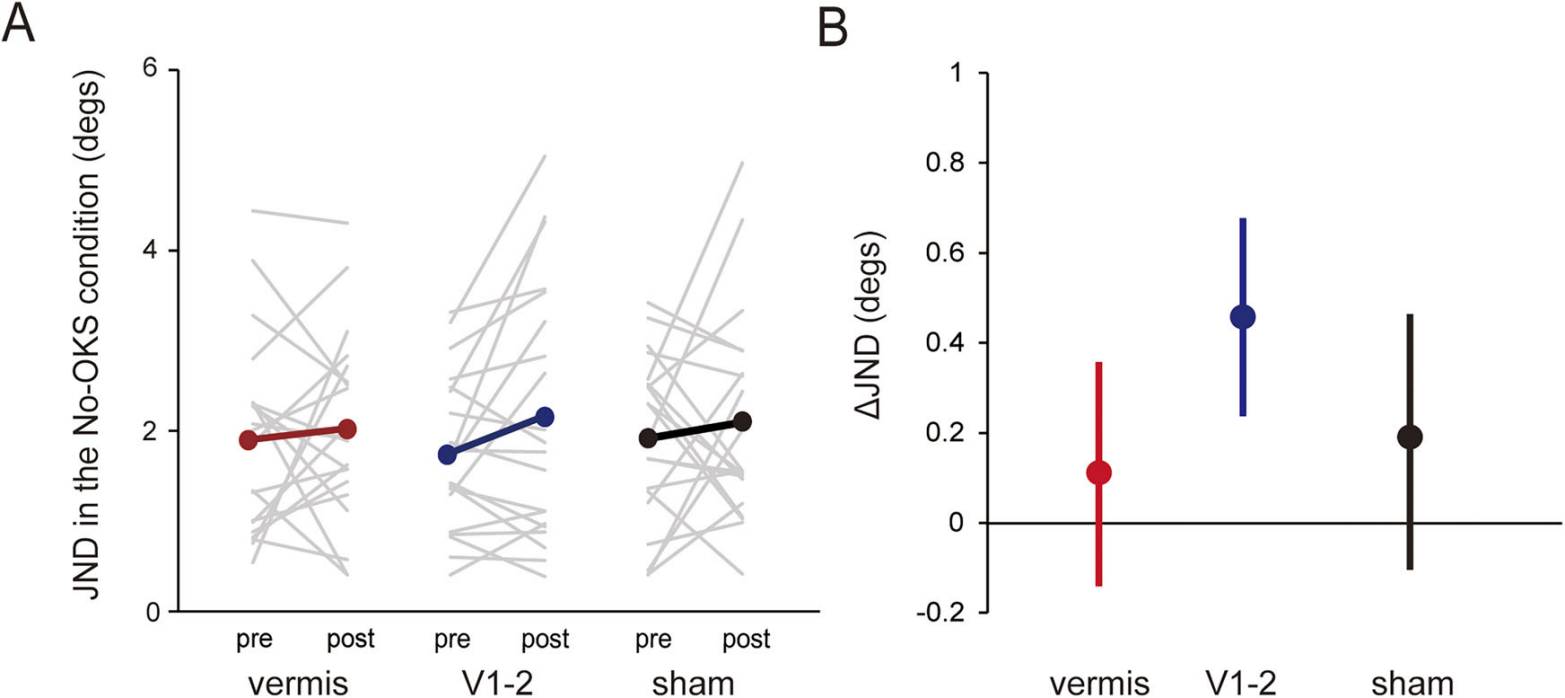
***A***, JND in the No-OKS condition for each phase and rTMS condition. Gray thin lines and colored (red, vermis; blue, V1–2; black, sham) thick lines represent individual and group mean values, respectively. ***B***, ΔJND for each rTMS condition. The error bars represent the standard deviation. JND, just noticeable deference; OKS, optokinetic stimulation; rTMS, repetitive transcranial magnetic stimulation.

10.1523/ENEURO.0111-25.2025.f1-3 Figure 1-3 Estimated EF distribution (V/m) in the ventral region of the cerebellum under the vermis condition. The maximum and mean EF values within the ROI covering both flocculi were 24.1 V/m and 10.0 V/m, respectively. EF, electric field; ROI, region of interest. Download Figure 1-3, TIF file.

**Figure 4.**
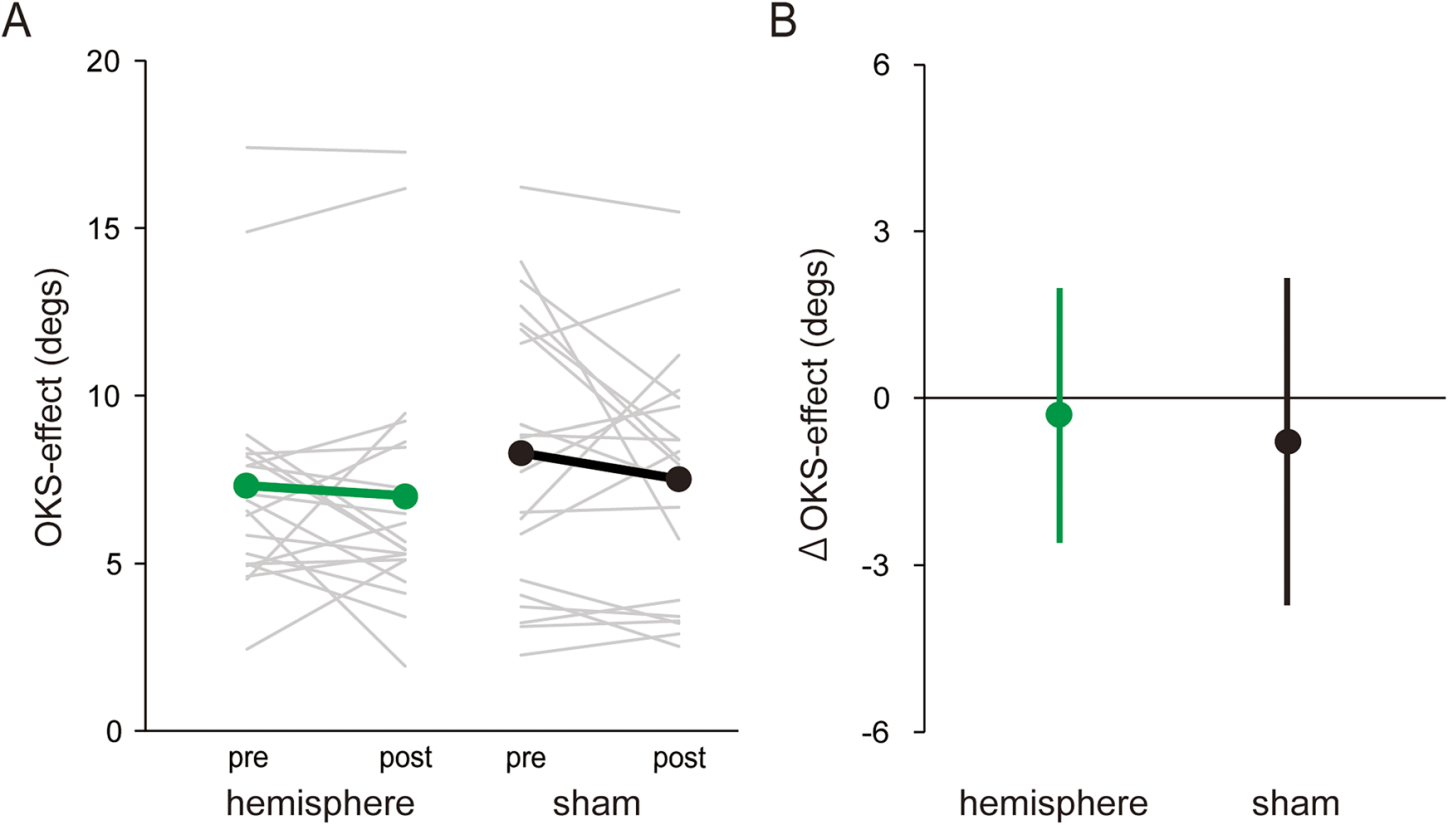
***A***, The OKS-effect on SVV judgment for each phase and rTMS condition in Experiment 2. Gray thin lines and colored (green, hemisphere; black, sham) thick lines represent individual and group mean values, respectively. ***B***, ΔOKS-effect for each rTMS condition. The error bars represent the standard deviation. SVV, subjective visual vertical; OKS, optokinetic stimulation; rTMS, repetitive transcranial magnetic stimulation.

10.1523/ENEURO.0111-25.2025.f1-4 Figure 1-4 Sagittal slice illustrating the EF distribution (V/m) in the vermis and V1–2 conditions. Each black dot represents the location of the maximum EF within each ROI. EF, electric field; ROI, region of interest. Download Figure 1-4, TIF file.

10.1523/ENEURO.0111-25.2025.f1-5 Figure 1-5 Depth-dependent changes in EF strength under the vermis condition. The horizontal axis indicates the depth (in mm) from the coil surface, which approximately corresponds to the scalp. Gray lines represent the EF strength in individual head models, whereas the black line shows the mean EF across the 18 models. The mean ± standard deviation penetration depth, defined as the depth at which the EF strength decreases to half of its value at the coil surface, was 19.7 ± 10.6 mm. EF, electric field. Download Figure 1-5, TIF file.

10.1523/ENEURO.0111-25.2025.f1-6 Figure 1-6 The estimated EF distribution (V/m) for the coil placement (1cm below and right to the inion) assigned to the hemisphere condition (Experiment 2). EF, electric field. Download Figure 1-6, TIF file.

In each experiment, the participants were equally and randomly assigned to either the clockwise (CW) or counter-clockwise (CCW) groups. For the CW and CCW groups, CW and CCW OKS were provided in the OKS condition (see below, SVV judgment task) across all phases and rTMS conditions, respectively. This design allowed a reduction in the number of trials, which helped avoid lengthy experimental times for each participant.

### SVV judgment task

The participants sat on a chair with a head-mounted display (HMD; Oculus Rift S; Meta), placed their chin on the chin rest, and pressed the superior anterior portion of the HMD against the forehead rest combined with the chin rest during the sessions (Fig. 1*B*). A nonslip tape was attached between the HMD and forehead rest to minimize involuntary head movements that can be induced by OKS. The head tilt angle in the frontal plane was continuously monitored via the HMD device, and if the head angle was larger than 1° in the CW or CCW direction during the SVV judgment task, the experimenter manually adjusted the participant's head to an upright position. During the task session, white noise was provided through the HMD to avoid the surrounding auditory cues.

In each SVV task session, the No-OKS condition was initiated by the appearance of an annulus of multicolored (green, white, blue) stationary dots, serving as visual background [outer diameter, 105.5°; inner diameter, 17.9° (viewing angle); density, 76.9 dots/m^2^], along with a gray fixation dot [diameter, 0.9° (viewing angle)] in the center of display for 10 s (Fig. 1*A*, botom panel, initial dot presentation). Instead of the fixation point, a virtual line consisting of a row of seven gray dots [each dot size, 0.9°; total length of the line, 8.1° (viewing angle)] was presented for 200 ms at a certain tilt angle (Fig. 1*A*, bottom panel, line presentation). Dots were used instead of a solid line to avoid orientation cues that could arise from the visual aliasing of a solid line (Dakin et al., 2018). The tilt angle of the visual line was randomly selected from a set of 12 angles: ±16.5°, ±8.5°, ±4.5°, ±2.5°, ±1.5°, and ±1° relative to the vertical. Positive and negative signs indicate the CW (rightward) and CCW (leftward) directions, respectively. Then, a gray fixation point appeared in the center for 2 s (Fig. 1*A*, bottom panel, judgment), within which the participant judged whether the line orientation was tilted in the CW or CCW direction with respect to the gravitational vertical using a two-alternative forced-choice paradigm by pressing one of two keys on a customized numeric keypad (TK-TCM011SV; Elecom) with the right and left thumbs. Participants were also asked to avoid repressing the button during this phase; if they pressed the button, the last button pressed was recorded as the judgment. After the fixation point had disappeared, the visual line was presented again at a certain angle for the next trial. Each line angle was presented twice per block, resulting in 24 trials per block. This block was repeated four times in the No-OKS condition, for a total of 96 trials.

After all trials in the No-OKS condition were completed, the OKS condition was started. Prior to the presentation of the virtual line for the SVV judgment task, the visual background (annulus of dots) was rotated in the CW or CCW direction (depending on the participant's group) with a center fixation point for 30 s (“Initial dot presentation”). This pre-exposure period provides a stable assessment of the effect of OKS on SVV performance, considering a previous study (Dakin et al., 2018) that showed that the SVV bias toward OKS increased with exposure time but reached a saturation point at 30 s. The angular velocity of the OKS was 30°/s, which could effectively shift the SVV in the direction of the OKS (Dichgans et al., 1972). The SVV trial was then started, with 96 trials completed under the OKS condition and with the same procedure as in the No-OKS condition. Before the SVV task session, the participants were instructed to perform the SVV judgment task without paying attention to the visual background. The total duration of each SVV judgment task session was ∼10 min.

### TMS

#### Stimulus parameter

In each rTMS session, low-frequency (1 Hz) rTMS was applied to the target cortical site (see below) for 15 min (i.e., 900 pulses) using a MagPro R20 magnetic stimulator (MagVenture) with a double-cone coil (D-B-80; MagVenture). The double-cone coil is designed for deeper brain stimulation, providing a broader magnetic field distribution that facilitates penetration into deep midline structures (Gomez-Tames et al., 2020) including the cerebellar vermis. We selected 1 Hz rTMS over continuous theta-burst stimulation; although they can produce similar levels of inhibition (Zafar et al., 2008), 1 Hz rTMS has a well-established safety profile and a lower risk of side effects (Rossi et al., 2009, 2021; Cole et al., 2024).

The stimulus intensity of rTMS in each experiment was determined according to each individual's resting motor threshold (rMT; Cattaneo et al., 2014; Fiori et al., 2015), which was measured on the first experimental day before the experiment. In the present study, we applied rTMS at a fixed intensity across the experimental days, rather than adjusting it according to the daily rMT (Gorsler et al., 2003), because rMT, which reflects corticospinal excitability, may not appropriately capture daily fluctuations in the excitability of non-motor regions such as the cerebellum and visual cortex. The electromyographic signal of the right first dorsal interosseous muscle was acquired via Ag/AgCl surface electrodes, amplified, bandpass filtered between 15 and 3 kHz with an amplifier (MEG-1200; Nihon Kohden), and sampled at a rate of 10 kHz using a PowerLab 800S A/D converter (ADInstruments). The motor hotspot, i.e., the cortical spot where TMS produced the maximum amplitude of motor-evoked potentials in the right first dorsal interosseous muscle, was explored by delivering single-pulse TMS to various positions around the left primary motor area. rMT was defined as the minimum intensity that evoked motor-evoked potentials >50 µV for at least 5 of 10 stimulations applied to the motor hotspot during resting (Rossini et al., 2015). Concerning the coil orientation for rMT determination, the handle pointed backward and laterally at a 45° angle to the nasion–inion line (Mills et al., 1992). The intensity of TMS stimulation was set to 90% of the rMT for each participant. The mean (± standard deviation) intensity was 34.0 ± 5.7% of the maximum stimulator output.

#### Determination of coil placement

In Experiment 1, two stimulation sites were targeted: the cerebellar vermis (vermis condition) and the V1–2 regions of the visual cortex (V1–2 condition). The neuronavigation system and individual head magnetic resonance imaging (MRI) were not used to target the brain regions. Instead, we simulated the electric fields (EFs) induced by TMS using high-resolution generic head models at the group level, with the anatomical landmark (inion) serving as the reference point for coil placement. Coil placement was determined on the basis of the simulation results (see below).

The head models were constructed with a spatial resolution of 0.5 mm, derived through the segmentation of T1- and T2-weighted structural MR images of 18 male participants (21–55 years) were obtained from a freely available repository (NAMIC: Brain Multimodality, 3.0 T MRI scanner, 1 mm voxel size, available online at http://hdl.handle.net/1926/1687). A detailed explanation of head models can be found in our previous study (Laakso et al., 2015). Each segmented head model included detailed anatomical structures, with electrical conductivities assigned to individual tissue types (e.g., gray matter, white matter, cerebrospinal fluid, and bone) based on established literature values (Gabriel et al., 1996). The development and validation of these models followed the procedures detailed in previous studies (Laakso et al., 2015; Laakso et al., 2018). For volume conductor analysis, the scalar potential finite difference method (Dawson and Stuchly, 1997), with a multigrid algorithm (Laakso and Hirata, 2012), was used to compute the induced EF. The electrical conductivity was assigned to each tissue based on the values provided by Gabriel et al. (1996). This approach calculates the EF distribution by solving the quasi-static Maxwell equations with the vector potential of the TMS coil as the input parameter. A simulation was executed for each head model to predict site-specific EF distributions under the given coil position. As a postprocessing step, the EF values from each personalized head model were transformed into a standard brain space, allowing for analysis at the group level. The stimulation sites, the cerebellar vermis, and V1–2 regions were defined anatomically based on standard brain atlases and functional relevance to the experimental objectives (Fischl, 2012).

To determine the coil placement, points were first marked along the inion–nasion line of the head model between 2 cm below and 5 cm above the inion at 0.5 cm intervals. For the cerebellar vermis, the region of interest (ROI) was set in the vermis VII region, which is functionally implicated in both visual motion (Cattaneo et al., 2014) and vestibular processing (Stephan et al., 2005). Although ventral (caudal) areas of the vermis, such as the flocculus, also contribute to vestibular processing and optokinetic responses (Waespe et al., 1983), these deep regions were not targeted in the present study because of the difficulty in stimulating them using TMS (Extended Data Fig. 1-3). For the V1–2 region, ROI was set in the combined region of the lingual gyrus (Brodmann 17 area) and cuneus (Brodmann 18 area). The maximum and mean EF values in each ROI were calculated when the TMS coil was placed at each point (Extended Data Fig. 1-1). To maximize the focality of the stimulation, we selected the point 1.5 cm below the inion to stimulate the vermis, whereas the point 4 cm above the inion was stimulated in the V1–2 condition based on the EF data. We verified that these coil placements ensured a high EF at the target ROIs while keeping the EF at nontarget ROIs relatively low (Fig. 1*C*, Extended Data Fig. 1-2). The EF distribution appeared to be asymmetrical even though the coil was placed on the midline of the cortex, likely because of the coil design and underlying anatomy.

The coil orientation for stimulation was set as the handle was oriented upward in both the vermis and V1–2 conditions (Cattaneo et al., 2014). In the sham condition, the coil was set at the same position as in the vermis condition but was tilted backward at a 90° angle relative to the scalp surface (i.e., the handle was oriented backward), with one edge touching the scalp to reproduce the auditory click and somatosensory sensation without effective brain stimulation.

In Experiment 2, although the stimulation parameters were adapted from Experiment 1, the target stimulation site was the right cerebellar hemisphere (hemisphere condition). In this condition, the TMS coil was placed 1 cm inferior to the inion on an inion–nasion line and 3 cm lateral to the right, as in previous studies (Hardwick et al., 2014; Matsugi et al., 2024) targeting the cerebellum hemisphere. As in Experiment 1, we simulated the EFs induced by coil placement. EF simulation with the ROI set over the entire right cerebellar hemisphere showed that this coil placement induces a strong EF in the right hemisphere (mean EF, 18.0 V/m; maximum EF, 127.0 V/m), whereas the EFs in the vermis was weaker (mean EF, 26.4 V/m; maximum EF, 54.3 V/m) than that in the vermis condition (mean EF, 34.8 V/m; maximum EF, 68.3 V/m; Extended Data Figs. 1-1, 1-6). The coil orientation for this condition was identical to that of the vermis condition. In the sham condition, although the coil position was identical to that in the hemisphere condition, the coil surface was tilted backward at a 90° angle relative to the scalp surface (i.e., with the handle oriented backward), as in Experiment 1.

### Data analysis

Responses (CW or CCW) were recorded for each trial of the SVV judgment task. If there was no response within the judgment time limit (2 s), the trial was excluded from further analysis.

The proportion of CW responses in each line orientation was calculated for each phase and the rTMS condition. The psychometric function was estimated by fitting a cumulative Gaussian function to each individual's response data using the Psignifit 4 toolbox (Schütt et al., 2016) in MATLAB (MathWorks). Based on the computed psychometric function, we calculated the SVV and judgment precision values as indices of task performance (see below for details).

#### SVV

The SVV value was determined as the angle at which the visual line was equally likely to be judged as tilted in the CW or CCW direction with respect to the gravitational vertical. Positive and negative values of the SVV indicated deviation in the CW and CCW direction, respectively. Using SVV data, we performed the following steps to quantify the effect of rTMS on visual dependency for each participant. First, the effect of the OKS on the SVV value (OKS-effect) was calculated for each phase by subtracting the SVV value in the No-OKS condition from that in the OKS condition. Next, the positive–negative sign of the OKS-effect was reversed only for the participants in the CCW group, so that a positive OKS-effect value indicated a shift of SVV toward the direction of OKS (i.e., a larger OKS-effect value indicates higher visual dependency) for all participants. We confirmed that the OKS-effect was not significantly different between groups in all phases and rTMS conditions in either experiment (Student's *t* test, all *p* > 0.05), indicating that the magnitude of the OKS-effect did not depend on the rotational direction of the OKS (i.e., participant group). Finally, ΔOKS-effect, an indicator of the effect of rTMS on visual dependency, was calculated by subtracting the OKS-effect in the pre-rTMS phase from that in the post-rTMS phase for each rTMS condition.

#### Judgment precision

The cerebellum, including the vermis, has been shown to contribute to the processing of vestibular signals (Bronstein et al., 2008; Bertolini et al., 2012; Dahlem et al., 2016; see also Barmack, 2003 for a review), and cerebellar rTMS may therefore influence vestibular processing. The maximum likelihood estimation (MLE) framework assumes that the brain determines the weight of each sensory signal when integrating them according to their reliability, which is the inverse of sensory noise or uncertainty (Ernst and Banks, 2002; Angelaki et al., 2009); that is, the more reliable the cues, the greater the sensory weight assigned to them.

According to this framework, if cerebellar rTMS increases or decreases the noise (i.e., alters the reliability) of vestibular signals during visual vertical judgments, it could result in a corresponding change in the relative visual weight; that is, visual dependency. Although visual vertical estimation also involves other modalities, such as somatosensory (proprioceptive) information, a previous study showed that its precision is predominantly reflected in vestibular (otolith) signals (Tarnutzer et al., 2009). In support of this, SVV imprecision when the body is upright has been reported in patients with vestibular disorders (Alberts et al., 2018; Bürgin et al., 2018), whereas no such impairment has been observed in somatosensory disorders (Anastasopoulos and Bronstein, 1999). Furthermore, other studies suggested that the brain assigns much greater sensory weight to otolith signals than to bodily (somatosensory) signals when estimating gravitational direction at the near-upright position (Alberts et al., 2016). These findings suggest that, at least when the head/body is upright, visual vertical estimation in the absence of visual background cues predominantly relies on vestibular (otolith) processing.

Therefore, to assess the effect of rTMS on vestibular noise underlying the estimation of visual vertical, we quantified the precision in the SVV judgment task by calculating just-noticeable difference (JND) under the No-OKS condition and evaluated how it was affected by cerebellar rTMS.

We first calculated the stimulus values (i.e., line angles) at a response rate of 75% $\left(LA_{75}\right)$ on the basis of the fitted psychometric function. Because the fitted function was symmetric, the JND for each phase and rTMS condition was determined as the difference between the line angle at a response rate of 50% (i.e., SVV) and the $LA_{75}$ (Gescheider, 1997; Rösler et al., 2015; Yao et al., 2019). In this symmetric case, the JND can also be considered as an index of the slope of the psychometric function around the SVV point, with larger JNDs indicating less precise perceptual judgments. As in the SVV task, the effect of rTMS on the JND (ΔJND) was computed by subtracting the JND in the pre-rTMS phase from that in the post-rTMS phase.

#### Statistical analysis

In Experiment 1, data from two participants were excluded from the group analysis because of significant data loss (∼1/3 of all trials; ID: 9) and noncompletion of the experiment because of nausea (although without seizures or syncope) during OKS exposure (ID: 13). After excluding trials with no response within the time limit (2 s), 99.3% of the total trials were included in the group analysis. In Experiment 2, no participants were excluded. After removing trials without responses, 99.3% of the total trials were retained for the group analysis.

To evaluate the group effect of rTMS on the cerebellar vermis or V1–2 on each judgment performance measure (ΔOKS-effect, ΔJND), we conducted statistical analysis using a linear mixed-effect model (LMEM) for each performance using the lme4 package (Bates et al., 2015) implemented in R (version 4.3.3; R Development Core Team). The model for Experiment 1 is as follows:

lmer(Perf∼Vermis+V1−2+PrePerf+(1|Participant)+(1|Group)),

where Perf represents each performance metric (ΔOKS-effect or ΔJND) as the dependent variable and Vermis and V1−2 are binary-coded predictors indicating the rTMS condition. Specifically, the Vermis term was coded as 1 when rTMS was applied to the vermis and as 0 otherwise, whereas the V1−2 term was coded as 1 when rTMS was applied to V1–2 and as 0 otherwise. The sham condition was represented by 0 for both terms, serving as the reference condition. We also included each performance in the pre-rTMS phase (i.e., OKS-effect_pre_ or JND_pre_, denoted in the equation by PrePerf) into the model as a fixed effect to control for the effect of baseline performance before rTMS exposure, which may affect the effect of rTMS on performance (Corp et al., 2020). Additionally, two factors, participant and group (CW or CCW), were included as random intercepts ( Participant, Group).

As mentioned above, an LMEM analysis was also performed for ΔOKS-effect in Experiment 2. In this model, two fixed-effect factors, rTMS over the cerebellar hemisphere (Hemisphere) and OKS-effect in the pre-rTMS phase (OKS-effect_pre_), and two random intercepts (Participant,Group) were included. The effect size $\left(\eta_{p}^{2}\right)$ of each factor in the LMEM was calculated. The significance level for all statistical analyses was set at *p* < 0.05.

## Results

No participants in either Experiment 1 or Experiment 2 showed any TMS-induced side effects such as neck muscle pain, headaches, or other discomfort.

### Experiment 1

#### SVV

Figure 2*A* shows the proportion of CW responses at each line angle and the fitted psychometric functions of a representative participant (ID: 3; CW group) for each OKS condition and the rTMS phase in the sham condition. The SVV value was nearly zero in the No-OKS condition for both rTMS phases (pre-rTMS phase, −0.73°; post-rTMS phase, −2.18°), indicating accurate estimation of the visual vertical without visual rotational cues. In the OKS condition, the proportion of responses opposite (i.e., CCW) to the OKS direction increased, leading to SVV shifts toward the OKS (i.e., CW) direction (pre-rTMS phase, 4.19°; post-rTMS phase, 4.04°). This shift was confirmed at the group level by the positive (CW group) and negative SVV angles (CCW group), regardless of the rTMS condition or phase (Table 1).

**Table 1.** Mean SVV (degrees) of CW and CCW groups for each rTMS condition and phase in Experiment 1

		Vermis		V1		Sham	
		Pre	Post	Pre	Post	Pre	Post
CW group	No-OKS	−1.37 (2.52)	−0.71 (2.26)	−1.57 (1.16)	−0.89 (1.93)	−1.33 (2.32)	−1.25 (3.32)
	OKS	5.06 (5.52)	4.64 (3.17)	4.36 (3.91)	5.44 (5.04)	4.53 (4.94)	5.09 (5.21)
CCW group	No-OKS	0.05 (1.76)	0.19 (1.95)	0.86 (1.86)	1.03 (2.11)	−0.24 (1.77)	0.02 (1.45)
	OKS	−4.83 (3.28)	−4.30 (3.90)	−4.47 (3.24)	−4.10 (4.94)	−5.25 (3.74)	−5.99 (4.46)

The values in parentheses represent the standard deviation. CW, clockwise; CCW, counter-clockwise; OKS, optokinetic stimulation.

Although a positive OKS-effect (i.e., SVV shifts to the OKS direction) was observed for all rTMS conditions and phases, the OKS-effect tended to decrease after rTMS exposure in the vermis condition, whereas a slight change or increased OKS-effect was observed in the V1–2 and sham conditions (Fig. 2*B*), as shown by ΔOKS-effect (vermis, −0.73 ± 2.66°; *V*1–2, 0.10 ± 2.07°; sham, 0.74 ± 2.30°; Fig. 2*C*). The LMEM revealed a significant negative effect of Vermis on ΔOKS-effect (*β* = −1.39, *t* = −0.10, *p* = 0.33, $\eta_{p}^{2}$ = 0.03), whereas the effect of V1−2 was not significant (*β* = −0.56, *t* = −0.10, *p* = 0.33, $\eta_{p}^{2}$ = 0.03). There was a significant negative coefficient for the OKS-effect_pre_ (*β* = −0.40, *t* = −3.82, *p* < 0.001, $\eta_{p}^{2}$ = 0.28; Fig. 2*D*), implying that participants with a larger OKS-effect in the pre-rTMS phase showed a smaller ΔOKS-effect. These results indicate that visual dependency in the SVV judgment task was attenuated by 1 Hz rTMS over the cerebellar vermis.

Additionally, we checked whether the OKS-effect in the pre-rTMS condition (i.e., OKS-effect_pre_) differed across rTMS conditions using another LMEM with two separate fixed-effect factors ( Vermis and V1−2) and a random-effect factor (participant). No significant effects of Vermis (*β* = 0.22, *t* = 0.41, *p* = 0.69, $\eta_{p}^{2}$ = 0.00) or V1−2 (*β* = 0.20, *t* = 0.36, *p* = 0.72, $\eta_{p}^{2}$ = 0.00) were observed, indicating no significant difference in the OKS-effect_pre_ between the rTMS conditions.

Despite the significant effect of vermis rTMS, the ΔOKS-effect in the sham condition was >0. This result suggests that the significant difference in ΔOKS-effect between the vermis and sham conditions might have been caused by a spurious increase in the OKS-effect in the sham condition rather than the effect of rTMS on the vermis. Considering this possibility, we also checked whether a significant change in OKS-effect occurred before and after rTMS exposure in each rTMS condition by conducting additional statistical analysis using the LMEM, with OKS-effect as the dependent variable. As for ΔOKS-effect, Vermis and V1−2 were incorporated as fixed effects and Participant and Group as random intercepts. In addition, a Phase term (coded as 0 for pre-rTMS and 1 for post-rTMS) and interaction terms between Phase and Vermis or V1−2 were included as fixed effects. There was a marginal negative interaction between Phase and Vermis (*β* = −1.48, *t* = −1.94, *p* = 0.055, $\eta_{p}^{2}$ = 0.04) but no significant effects of Vermis (*β* = 0.22, *t* = 0.42, *p* = 0.68, $\eta_{p}^{2}$ = 0.02), V1−2 (β = 0.20, *t* = 0.37, *p* = 0.71, $\eta_{p}^{2}$ = 0.00), Phase (*β* = 0.74, *t* = 1.38, *p* = 0.17, $\eta_{p}^{2}$ = 0.00), or the Phase and V1−2 interaction (*β* = −0.64, *t* = −0.85, *p* = 0.40, $\eta_{p}^{2}$ = 0.00). Although this effect was marginal, it suggests that OKS-effect specifically decreased after rTMS was applied to the vermis.

We also examined whether rTMS influenced accuracy in the SVV judgment task without visual rotational cues (i.e., SVV in the No-OKS condition). We calculated the change in SVV (ΔSVV) in the No-OKS condition between the phases by subtracting the SVV value in the pre-rTMS phase from that in the post-rTMS phase. ΔSVV was 0.40 ± 1.43° in the vermis condition, 0.43 ± 1.18° in the V1–2 condition, and 0.17 ± 1.39° in the sham condition. LMEM analysis with three fixed effects ( Vermis V1−2, and SVV in the pre-rTMS phase) and two random intercepts ( Participant, Group) showed no significant effect of vermis rTMS (*β* = 0.36, *t* = 1.07, *p* = 0.29, $\eta_{p}^{2}$ = 0.03) or V1–2 rTMS (*β* = 0.38, *t* = 1.12, *p* = 0.27, $\eta_{p}^{2}$ = 0.03), with no significant SVV coefficient in the pre-rTMS phase (*β* = −0.07, *t* = −0.79, *p* = 0.43, $\eta_{p}^{2}$ = 0.02). Thus, rTMS to the vermis apparently did not affect the accuracy of the SVV judgment task in the absence of visual rotational cues, consistent with the clinical finding (Tarnutzer et al., 2011) that patients with vestibulo-cerebellar degeneration showed accurate estimation of visual vertical.

#### JND

Figure 3*A* shows the JND in the No-OKS condition for each rTMS condition and phase. The JNDs tended to slightly increase (i.e., decrease in precision) after rTMS exposure in all rTMS conditions. For the ΔJND, LMEM analysis revealed that the effects of Vermis (*β* = −0.33, *t* = −0.32, *p* = 0.74, $\eta_{p}^{2}$ = 0.00) and V1−2 (*β* = 0.47, *t* = 0.47; *p* = 0.64, $\eta_{p}^{2}$ = 0.00) were not significant (Fig. 3*B*). The negative coefficient of $\;\mathrm{JN}D_{\mathrm{pre}}$ was significant (*β* = −0.70, *t* = −5.13; *p* < 0.001, $\eta_{p}^{2}$ = 0.32).

To further examine the effects of rTMS, we compared JNDs in the No-OKS condition between the pre- and post-rTMS phases using another LMEM analysis with Vermis, V1−2, Phase, and their interaction as fixed effects and Participant and Group as random intercepts. No significant fixed-effect factors were found (all at *p* > 0.47, $\eta_{p}^{2}$ ≤ 0.02). Together, these results suggest that precision in visual vertical judgments in the absence of visual rotational cues may not be affected by rTMS over the cerebellar vermis, in contrast to visual dependency.

To confirm whether the OKS influenced judgment precision, we quantified the OKS-effect on the JND (termed OKS-effect_JND_) by subtracting the JND in the No-OKS condition from that in the OKS condition. OKS-effect_JND_ was positive regardless of phase and rTMS condition (mean ± standard deviation: vermis, 1.90 ± 1.24° and 1.43 ± 0.65°; V1–2, 2.51 ± 1.54° and 2.09 ± 2.57°; sham, 1.95 ± 0.95° and 1.01 ± 0.67° in the pre- and post-rTMS phase, respectively). These results indicate that visual motion cues reduced the precision of visual vertical judgments, consistent with previous findings [Ward et al., 2017 (trend only); Dockheer et al., 2018].

### Experiment 2

Similar to Experiment 1, SVV was nearly zero in the No-OKS condition, whereas SVV was strongly biased toward the direction of OKS in the OKS condition regardless of the rTMS condition or phase (Table 2), supported by the positive OKS-effect (Fig. 4*A*).

**Table 2.** Mean SVV (degrees) of CW and CCW groups for each rTMS condition and phase in Experiment 2

		Hemisphere		Sham	
		Pre	Post	Pre	Post
CW group	No-OKS	0.24 (1.97)	−0.13 (1.55)	0.56 (2.00)	0.91 (3.17)
	OKS	8.37 (4.98)	7.35 (5.43)	8.62 (5.29)	8.81 (5.61)
CCW group	No-OKS	0.42 (1.74)	0.05 (2.21)	0.30 (2.53)	−0.40 (2.33)
	OKS	−6.09 (2.31)	−6.49 (2.68)	−8.25 (4.27)	−7.55 (4.41)

The values in parentheses represent the standard deviation. CW, clockwise; CCW, counter-clockwise; OKS, optokinetic stimulation; rTMS, repetitive transcranial magnetic stimulation.

The mean ΔOKS-effect decreased in the post-rTMS phase for both rTMS conditions (−0.31 ± 2.29° for the hemisphere condition, −0.78 ± 2.94° for the sham condition; Fig. 4*B*). The LMEM revealed no significant effect of Hemisphere (*β* = 0.53, *t* = 0.67, *p* = 0.51, $\eta_{p}^{2}$ = 0.02) on ΔOKS-effect for the SVV task with no significant coefficient of OKS-effect_pre_ (*β* = −0.17, *t* = −1.56, *p* = 0.13, $\eta_{p}^{2}$ = 0.10). As in Experiment 1, we also checked whether the change in the ΔOKS-effect between the phases was significant for each rTMS condition using another LMEM with two fixed factor ( Hemisphere, Phase), one fixed interaction (Hemisphere×Phase), and two random factors ( Participant, Group). There were no significant effects of Hemisphere (*β* = −0.98, *t* = −1.48, *p* = 0.14, $\eta_{p}^{2}$ = 0.04) and Phase (*β* = −0.78, *t* = −1.17, *p* = 0.25, $\eta_{p}^{2}$ = 0.02) and interaction (*β* = 0.48, *t* = 0.51, *p* = 0.62, $\eta_{p}^{2}$ = 0.00). These results indicate that rTMS over the right cerebellar hemisphere did not affect visual dependency for the SVV judgment.

## Discussion

In the present study, to confirm the direct involvement of the cerebellum in the visuo-vestibular interaction for the perception of gravitational vertical, we investigated the effect of 1 Hz rTMS over the posterior cerebellum on visual dependency, quantified by SVV bias induced by rotating OKS. A previous finding that patients with cerebellar degeneration (SCA6) showed exaggerated visual dependency (Dakin et al., 2018), we hypothesized that 1 Hz inhibitory rTMS over the cerebellum could enhance visual dependency in relation to SVV judgment. Contrary to our hypothesis, the OKS-induced SVV bias was attenuated after rTMS exposure of the cerebellar vermis. This attenuation was not observed after rTMS over adjacent areas, such as the early visual cortices (Experiment 1) and the cerebellar hemisphere (Experiment 2). Moreover, rTMS to the vermis had no significant effect on judgment precision in the absence of visual motion cues, which is likely to be based on vestibular processing.

Consistent with previous findings (Dichgans et al., 1972; Ward et al., 2017; Dakin et al., 2018; Niehof et al., 2019), the rotating OKS in this study led to a strong SVV bias in the direction of rotation, confirming that the brain integrates vestibular otolith and visual motion cues to estimate the gravitational direction (see Dakin et al., 2018 for a review). Importantly, our finding that the OKS-induced SVV bias was attenuated specifically in the vermis condition (Fig. 2) indicates that the cerebellar vermis contributes to visuo-vestibular interactions in relation to the perception of gravitational direction. Animal studies have demonstrated that Purkinje cells in parts of the caudal cerebellar vermis (such as the nodulus and uvula), referred to as the vestibular cerebellum, respond not only to vestibular stimulation but also to visual motion stimulation (Kano et al., 1991; Yakusheva et al., 2013). Human studies have also shown that the cerebellar vermis is activated during visually induced self-motion illusions (Becker-Bense et al., 2012). Furthermore, some neurons in the fastigial nucleus of macaques, which receive inputs from the nodulus/uvula and vermis, were found to respond to OKS (Bryan and Angelaki, 2009).

Dakin et al. (2018) interpreted the increased visual weight (dependency) in patients with SCA as an indirect consequence of the reduced reliability (i.e., increased noise) of vestibular otolith signals, given that an MLE model assuming increased vestibular noise because of a cerebellar deficit, without any effect on visual noise, fitted SVV data from patients with SCA. If inhibitory rTMS to the cerebellum increases vestibular noise, but does not affect visual noise, it should lead to an increase in the visual weight in visual vertical estimation, as observed in patients with SCA. In contrast, OKS-induced SVV bias was attenuated in the vermis condition in this study, leading us to speculate that 1 Hz rTMS to the vermis might have increased the noise in visual motion signals. Previous studies have shown that the cerebellar vermis is directly involved in processing visual motion signals (Nawrot and Rizzo, 1995, 1998; Bense et al., 2006; Ignashchenkova et al., 2009; Cattaneo et al., 2014; Baumann et al., 2015). Specifically, lesions in the midline cerebellum impair the ability to discriminate the direction of visual motion in humans (Nawrot and Rizzo, 1995, 1998) and monkeys (Ignashchenkova et al., 2009). The cerebellar vermis is functionally connected to the visual network (Kellermann et al., 2012; Sang et al., 2012). In contrast to the OKS-induced SVV bias, we did not observe significant changes in judgment precision (ΔJND; Fig. 3) in the absence of visual motion cues in the vermis condition, implying that vermis rTMS is unlikely to have effectively modulated vestibular processing underlying visual vertical estimation. Together, these results suggest that 1 Hz rTMS over the vermis might have increased visual noise by interfering with visual motion processing, while having less effect on vestibular processing, ultimately leading to reduced visual weight in visuo-vestibular integration processes.

rTMS over the early visual cortices (V1–2 condition) did not significantly affect visual dependency in this study (Fig. 2*C*). These regions play an important role in visual motion processing, along with the cerebellar vermis, by projecting local motion signals to extraretinal regions such as the MT/V5 area (Shipp and Zeki, 1989). This is supported by the finding that disrupting V1 using TMS reduces the ability to discriminate the direction of translational visual motion (Laycock et al., 2007; Cattaneo et al., 2014). Therefore, one may argue that inhibitory rTMS of this region could reduce visual dependency by interfering with visual motion processing, as in the vermis condition. Nevertheless, the lack of a significant effect of the V1–2 rTMS condition is likely attributable to the use of “circular” visual motion. Deutschländer et al. (2004) evaluated brain activity while visually induced self-motion (vection) was perceived about the line of sight (i.e., rotation in the frontal plane) or along the axis (i.e., translation) using positron emission tomography. They found that V1–2 (Brodmann areas 17 and 18) was strongly activated during translational vection, but not during rotational vection, compared with the presentation of stationary dots (i.e., no visual motion cues). Furthermore, Donato et al. (2020) reported that rTMS delivered to V1–2 had no effect on the discrimination of the direction of rotation of a random dot in the frontal plane. These findings suggest that the early visual cortex may not contribute to the processing of circular visual motion, which may be responsible for the lack of a significant effect of V1–2 rTMS on visual dependency in this study.

The effect of rTMS on visual dependency must be discussed carefully, as the difference in the effect of OKS between the pre- and post-rTMS phases in the vermis condition was not significant in this study, although there was a trend toward significance (*p* = 0.054). This lack of significance may be attributed to the difficulty of effectively stimulating the vermis, which is anatomically hidden beneath the cerebellar hemispheres and is located deep in relation to the scalp (Voogd and Glickstein, 1998). In fact, the simulated EF value at the target area was low with coil placement in the vermis condition compared with that in the V1 condition (Fig. 1*C*, Extended Data Figs. 1-2, 1-4). This result reflects a limitation of cerebellar TMS in this study; the modulation of neural activity in the vermis may have been limited, potentially resulting in an insufficient effect of rTMS.

### Limitations

Several limitations of this study should be noted.

First, although the group-level analysis revealed a statistically significant (partially marginal) effect of rTMS over the vermis, the sample size was modest (*n* *=* 20 for each experiment), and interindividual variability was considerable. Thus, further studies with a larger sample size may be necessary to confirm the contribution of the cerebellar vermis to visuo-vestibular interactions.

Second, we did not employ individual MRI-based neuronavigation to guide coil placement. Instead, coil positions were determined using an anatomical landmarks (the inion), informed by EF simulations in a set of high-resolution generic head models. Although the vermis seems to be less morphologically variable across individuals compared with other brain regions, such as the motor cortex (Steele and Chakravarty, 2018; Eichert et al., 2021), this approach may still have introduced variability in effective targeting, particularly given the depth of the cerebellar vermis. However, it is also worth noting that even when neuronavigation systems are employed, non-negligible localization errors have been reported (Nieminen et al., 2022).

Finally, our findings do not show whether the cerebellar vermis plays a direct role in combining and weighing visual and vestibular cues per se. Such a direct role may be unlikely, as the MLE model, which assumes changes only in vestibular noise, was found to explain SVV data across individuals regardless of cerebellar integrity (Dakin et al., 2018). Presumably, other cortical areas may be candidates for visuo-vestibular integration, especially for the orientation perception (Cheng and Gu, 2018; Cullen, 2019 for reviews). Electrophysiological studies in monkeys have demonstrated that neurons in the dorsal region of the medial superior temporal area and ventral intraparietal area are tuned to the direction of self-motion on the basis of both vestibular and visual motion signals (Bremmer et al., 2002; Gu et al., 2006, 2016; Chen et al., 2011). Moreover, recent studies have shown that human homologues of the dorsal region of the medial superior temporal area and ventral intraparietal area may also be involved in visual- and/or vestibular-based self-motion perception (Schindler and Bartels, 2018; Schmitt et al., 2020). It is likely that visual and vestibular signals are processed via various cortical and subcortical areas, including the cerebellum, and then integrated into higher-stage cortical areas including these regions to construct an internal representation of the direction of gravity (Kheradmand and Winnick, 2017). Further research is needed to investigate in detail how these cortical areas and the cerebellum interact in the perception of gravitational direction.

### Conclusion

The present study showed that visual dependency in visual vertical judgments was attenuated specifically after 1 Hz rTMS over the cerebellar vermis. This finding, although contradicting previous results in patients with cerebellar degeneration (Dakin et al., 2018), supports the direct involvement of the cerebellum in visuo-vestibular interaction for the perception of gravitational direction.

## Synthesis

Reviewing Editor: Niko Busch, Westfalische Wilhelms-Universitat Munster Decisions are customarily a result of the Reviewing Editor and the peer reviewers coming together and discussing their recommendations until a consensus is reached. When revisions are invited, a fact-based synthesis statement explaining their decision and outlining what is needed to prepare a revision will be listed below. The following reviewer(s) agreed to reveal their identity: Amir Kheradmand, Alexander Tarnutzer.

# Reviewer 1

This manuscript examines the effect of inhibitory repetitive TMS over the cerebellar vermis in comparison to TMS over visual cortex, cerebellar hemisphere, and sham stimulation. Overall, the significant results are marginal and the TMS effect is variable among individuals. This raises the question whether the sample size for the effect of TMS is adequate? It is unclear whether neuronavigation was used for TMS application even though the target effect was simulated. Was this simulation of TMS effect done in individual subjects or a generic model was used? It is confusing that it seems like MRIs from a 'freely available repository' was used instead of actual participants. Also, why lobule VII was targeted whereas areas such as flocculus are involved in OKN are more caudal than this targeted area?

Other comments:

Materials &Methods

Ideally the rMT should be determined on each experiment day. Please clarify how this was done.

Why is there asymmetry in the simulated EF at the target ROIs as shown in Fig 1C? Does this mean only one hemisphere was stimulated? If so, how was the decision made regarding which side to target?

There is no Extended Data Figure 1-1

Line 228-230, should the handle be oriented backward in the sham condition as noted earlier?

Lines 267-269, SVV is a multisensory measure and its precision cannot be only indicative of noise from otolith organs.

What's the reason that you chose 1 cm inferior to the inion and 3 cm lateral to the right to stimulate the cerebellar hemisphere? Does use of these anatomical landmarks mean that there was no use of neuronavigation or individual brain MRIs to apply TMS at specific target regions in this study?

Will the TMS of double cone coil reach comparable depth on the three stimulation locations?

Did not find JND in the paper (Barnett Cowan et al., 2010) you cited. If your fitted psychometric function is symmetric, (*LA*75−*SVV*) should be same as (*SVV*− *LA*25). The equation for JND can be simplified as (*LA*75−*SVV*). You can call this slope, but not appropriate as JND.

Did you calculate the effect of OKS on SVV precision or just compare precision in the No-OKS condition pre- and post-TMS?

Results

Suggest moving the first paragraph to Methods.

Line 312, please keep Sub ID number here consistent with the figure 2A legend.

Line 314 and line 317, make sure the numbers match the figure.

In Figure 2A, in pre-rTMS plot OKS condition, the proportion of CW response of one circle is about 0.25 but its neighbor circles are zero. So this could be caused by a couple of trials during which the participant pressed the wrong button. In post-rTMS plot, the line angle goes beyond the range you tested.

Can you do a separate correlation analysis between ΔOKS-effect and OKS-effectpre for three rTMS conditions? Any significant differences in OKS-effectpre among three rTMS conditions?

Line 326-327, this sentence is confusing. Please correct.

What's the effect of OKS on SVV precision?

Line 347-348, please clarify the coefficient of JND Pre

# Reviewer 2

General comment

In this original contribution the authors addressed the role of the cerebellum in the visual-vestibular interaction for the perception of direction of gravity by use of repetitive transcranial magnetic stimulation (rTMS). Previously an increased visual dependence has been reported in patients with cerebellar degeneration. To further address these observations in a more standardized way, an experimental paradigm was proposed. Specifically, the authors applied rTMS to 42 healthy human subjects to different brain areas and measured the subjective visual vertical (SVV) bias induced by accompanying optokinetic stimulation (OKS). They targeted both the dorsal cerebellar vermis, the cerebellar hemisphere and the early visual cortex. Sham stimulation was included as well. The key result presented is that rTMS applied to the cerebellar vermis significantly attenuated the OKS-induced shift in the SVV bias. This was not the case when applying rTMS to either the visual cortex or the cerebellar hemisphere. The authors concluded that their findings suggest a direct involvement of the cerebellar vermis in the visuo-vestibular interaction underlying the perception of gravitational direction.

Overall, this experimental study sheds further light on the cerebellum's role in modulating the multisensory integration of visual and vestibular input for spatial orientation. Strengths of the study are the hypothesis-driven approach with a sound rationale for performing this study and the well-described methods. But also the statistical analysis and the interpretation of the results are straight-forward and reasonable. Thus, I do not see any major issues. However, a few aspects require the authors' attention. This includes both the lack of neuro-navigation based on individual MR-imaging to identify the target locations and a discussion why rTMS was preferred over theta-burst stimulation.

Specific comments are provided below:

Abstract:

Line 7: please provide a more detailed location. cerebellar vermis? hemispheres??

Introduction:

Material and methods:

Line 99: please describe with more detail how the coil was placed in the sham condition. How remote was the coil from the surface of the skin or did it touch the skin but just had a perpendicular angle?

Line 140: the SVV paradigm used is often referred to an „alternate forced choice paradigm". It might be worthwhile referring to this for clarity.

Line 215: would it be possible to provide a sagital view in Fig 1C to illustrate the EF in the vermis. The reason is that the distance from the skin to the vermis can be several cm. Furthermore, a more technical description of the capabilities of the TMS coil used would be nice. Especially regarding ist capability to reach deeper brain areas.

Line 227: please provide information about estimated depth of penetration, currently only EF distribution on the cerebellar surface is shown.

Results

Line 308: please briefly provide information about side effects of TMS (not only for those that dropped out). Did you observe any neck (muscle) pain, headaches etc...?

Discussion:

In a limitations section the lack of individual neuro-navigation used to identify the target stimulation areas should be mentioned and potential downsides of using anatomical landmarks as the inion only should be discussed.

Probably the authors could also briefly discuss why they preferred 1Hz rTMS over continous theta-burst stimulation (cTBS)?

Figures

Figure 1: there is a typo in Fig 1A: task familiarzation instead of familiarization

# Synthesis

After careful evaluation by two experts in the field, we find that your study addresses an important question regarding the cerebellum's contribution to visuo-vestibular integration. Both reviewers appreciated the hypothesis-driven design and methodological rigor, including the use of multiple control sites. However, several important concerns need to be addressed, and I encourage you to carefully consider and respond to each of these points in your revision.

Both reviewers highlighted limitations related to the strength of the statistical evidence and the use of generic head models. I suggest following Reviewer 2's recommendation to add a "Limitations" section to the Discussion. This section should address, among others, the following points:

- A justification of the sample size, particularly in light of the small and variable effects observed;

- A clarification regarding the use of generic head models instead of individual MRIs, and the absence of neuronavigation, including a discussion of the potential limitations this entails.

## Author Response

# Reviewer 1 Comment 1 This manuscript examines the effect of inhibitory repetitive TMS over the cerebellar vermis in comparison to TMS over visual cortex, cerebellar hemisphere, and sham stimulation. Overall, the significant results are marginal and the TMS effect is variable among individuals. This raises the question whether the sample size for the effect of TMS is adequate? Response:

We are grateful to the reviewer for the thoughtful and valuable comments on our study. As the reviewer pointed out, the sample size for each experiment was modest (20 participants for each experiment). To address this, we employed within-subject comparisons and linear mixed-effects models to enhance the statistical power by accounting for individual variability. However, given that individual differences in the responsiveness to rTMS seem to be substantial (e.g., Hamada et al., 2013, Cereb Cortex), the modest sample size may have at least partially contributed to the variability in and marginal significance of our results. Therefore, further studies with larger sample sizes are needed to address this limitation and confirm direct involvement of the cerebellar vermis in visuo-vestibular interactions in association with the perception of gravitational direction. We have explicitly acknowledged this point in the revised Limitations section.

Changes:

Limitations First, although the group-level analysis revealed a statistically significant (partially marginal) effect of rTMS over the vermis, the effect size was modest (n = 20 for each experiment), and interindividual variability was considerable. Thus, further studies with a larger sample size may be necessary to confirm the contribution of the cerebellar vermis to visuo-vestibular interactions.

Comment 2 It is unclear whether neuronavigation was used for TMS application even though the target effect was simulated. Was this simulation of TMS effect done in individual subjects or a generic model was used? It is confusing that it seems like MRIs from a 'freely available repository' was used instead of actual participants.

Response:

We appreciate this comment.

Neuronavigation was not used in the present study. Instead, we employed electric field (EF) simulations using a set of 18 high-resolution anatomical models derived from a publicly available MRI repository to determine optimal coil placement. These simulations provided a generalized group-level estimation of the EF distribution, allowing us to define coil positions that target the cerebellar vermis and V1-2 regions with high focality. Note that approximately 20 models are shown to represent the group-level EF analysis (Laakso et al., 2015).

As we did not use participant-specific head models to estimate the EF, we did not perform individualized navigation-based targeting. Unlike motor cortex studies where inter-individual variability in cortical folding strongly affects the induced EF (Laakso et al., 2014), the cerebellar midline region (vermis) is likely less susceptible to large morphological variability across individuals (Steel et al., 2017).

These points have been clearly noted in the Materials and Methods and Limitations sections of the revised manuscript.

Changes:

Materials and Methods The neuronavigation system and individual head magnetic resonance imaging (MRI) were not used to target the brain regions. Instead, we simulated the electric fields (EFs) induced by TMS using high-resolution generic head models at the group level, with the anatomical landmark (inion) serving as the reference point for coil placement. Coil placement was determined on the basis of the simulation results (see below).

Limitations Second, we did not employ individual MRI-based neuronavigation to guide coil placement. Instead, coil positions were determined using an anatomical landmarks (the inion), informed by EF simulations in a set of high-resolution generic head models. Although the vermis seems to be less morphologically variable across individuals compared with other brain regions, such as the motor cortex (Eichert et al., 2016; Steel et al., 2017), this approach may still have introduced variability in effective targeting, particularly given the depth of the cerebellar vermis. However, it is also worth noting that even when neuronavigation systems are employed, non-negligible localization errors have been reported (Nieminen et al., 2022).

Comment 3 Also, why lobule VII was targeted whereas areas such as flocculus are involved in OKN are more caudal than this targeted area? Response:

We focused on lobule VII of the vermis because it is functionally implicated in both visual motion (e.g., Cattaneo et al., 2014) and vestibular processing (e.g., Stephan et al., 2005) in human studies. As the reviewer noted, ventral and caudal areas of the vermis, including the flocculus, also contribute to vestibular processing and optokinetic responses (e.g., Waespe et al., 1983, Exp Brain Res). However, noninvasively stimulating these deep brain areas with TMS is technically challenging, whereas lobule VII represents an accessible and well-validated target for examining cerebellar involvement in visuo-vestibular interactions.

To support this rationale, we conducted an additional electric field (EF) simulation to estimate EF values in the flocculus when the coil was placed in the same position as in the vermis condition. The results showed that the EF in the flocculus (mean: 10.0 V/m; maximum: 24.1 V/m) was substantially lower than that in the vermis (mean: 34.8 V/m; maximum: 68.3 V/m), confirming the difficulty of effectively stimulating deep cerebellar regions using TMS.

We have added a figure and the results for this additional EF simulation to the revised manuscript (Extended Data Fig. 1-1), along with a textual explanation of the rationale for not targeting ventral and caudal areas of the vermis, such as the flocculus.

Changes:

Materials and Methods For the cerebellar vermis, the region of interest (ROI) was set in the vermis VII region, which is functionally implicated in both visual motion (e.g., Cattaneo et al., 2014) and vestibular processing (e.g., Stephan et al. 2005). Although ventral (caudal) areas of the vermis, such as the flocculus, also contribute to vestibular processing and optokinetic responses (Waespe et al., 1983), these deep regions were not targeted in the present study because of the difficulty in stimulating them using TMS (Extended Data Figure 1-1).

Extended Data Figure 1-1 Estimated EF distribution (V/m) in the ventral region of the cerebellum under the vermis condition. The maximum and mean EF values within the ROI covering both flocculi were 24.1 V/m and 10.0 V/m, respectively. EF, electric field; ROI, region of interest.

Comment 4 Materials &Methods Ideally the rMT should be determined on each experiment day. Please clarify how this was done.

Response:

In this study, we applied rTMS at a fixed intensity for each participant on the basis of the resting motor threshold (rMT) measured on the first experimental day (i.e., 90% of rMT). We chose rMT because it is a widely accepted index for controlling inter-participant variability in cortical excitability. Although some TMS studies targeting M1 determined stimulus intensity on the basis of rMT measured on each experimental day (e.g., Gorsler et al., 2003), we did not adopt this approach because rMT can fluctuate daily within the same individual, not only because of changes in cortical sensitivity but also because of subtle differences in the experimental setup (e.g., hand posture, stimulation site, coil angle). Such fluctuations could introduce unnecessary intra-participant variability unrelated to the sensitivity of non-motor cortical areas, including the cerebellum. Moreover, to our knowledge, no clear evidence indicates that day-to-day changes in rMT reflect changes in cerebellar sensitivity to rTMS. Therefore, we believe using a fixed intensity across sessions can be considered a reasonable and consistent approach. We have added a description of the rMT measurement procedure to the text.

Changes:

Materials and Methods The stimulus intensity of rTMS in each experiment was determined according to each individual's resting motor threshold (rMT; Cattaneo et al., 2014; Fiori et al., 2015), which was measured on the first experimental day before the experiment. In the present study, we applied rTMS at a fixed intensity across the experimental days, rather than adjusting it according to the daily rMT (e.g., Gorsler et al., 2003), because rMT, which reflects corticospinal excitability, may not appropriately capture daily fluctuations in the excitability of non-motor regions such as the cerebellum and visual cortex.

Comment 5 Why is there asymmetry in the simulated EF at the target ROIs as shown in Fig 1C? Does this mean only one hemisphere was stimulated? If so, how was the decision made regarding which side to target? Response:

We appreciate these questions. Although coil placement was centered for vermis stimulation, slight asymmetry in EF is unavoidable, likely because of the coil design and underlying anatomy. This point is noted in the revised Materials and Methods section.

Changes:

Materials and Methods The EF distribution appeared to be asymmetrical even though the coil was placed on the midline of the cortex, likely because of the coil design and underlying anatomy.

Comment 6 There is no Extended Data Figure 1-1 Response:

Thank you for your comment.

We have confirmed that Extended Data Figures are included in the submitted files. Nevertheless, if there is an issue with how it is displayed, we will re-upload it.

Comment 7 Line 228-230, should the handle be oriented backward in the sham condition as noted earlier? Response:

We apologize for the incorrect description. As the reviewer indicated, the coil orientation for the sham condition was backward (not downward as in the hemisphere condition). We have corrected this error.

Changes:

Materials and Methods In the sham condition, although the coil position was identical to that in the hemisphere condition, the coil surface was tilted backward at a 90{degree sign} angle relative to the scalp surface (i.e., with the handle oriented backward), as in Experiment 1 Comment 8 Lines 267-269, SVV is a multisensory measure and its precision cannot be only indicative of noise from otolith organs.

Response:

As the reviewer pointed out, visual vertical estimation is derived from multisensory information, such as somatosensory (proprioceptive) signals, as well as vestibular signals. However, as already mentioned in the text, imprecision in SVV when the body was upright was found in vestibular disorders (Bürgin et al., 2018; Alberts et al., 2018), whereas SVV precision in the near-upright position was reported to be unaffected in somatosensory disorders (Anastasopoulos &Bronstein, 1999). In addition, some studies propose that the brain assigns much greater sensory weight to otolith signals compared with bodily (somatosensory) signals (Clemens et al., 2011; Alberts et al., 2016). Taken together, these findings suggest that, at least near the upright position, visual vertical estimation in the absence of surrounding visual cues may rely mainly on vestibular (otolith) processing. We have revised the relevant section of the manuscript to clarify this interpretation.

Changes:

Materials and Methods According to this framework, if cerebellar rTMS increases or decreases the noise (i.e., alters the reliability) of vestibular signals during visual vertical judgments, it could result in a corresponding change in the relative visual weight; that is, visual dependency. Although visual vertical estimation is also involves other modalities, such as somatosensory (proprioceptive) information, a previous study showed that its precision is predominantly reflected in vestibular (otolith) signals (Tarnutzer et al., 2009). In support of this, SVV imprecision when the body is upright has been reported in patients with vestibular disorders (Bürgin et al., 2018; Alberts et al., 2018), whereas no such impairment has been observed in somatosensory disorders (Anastasopoulos &Bronstein, 1999). Furthermore, other studies suggested that the brain assigns much greater sensory weight to otolith signals than to bodily (somatosensory) signals when estimating gravitational direction at the near-upright position (Clemens et al., 2011; Alberts et al., 2016). These findings suggest that, at least when the head/body is upright, visual vertical estimation in the absence of visual background cues predominantly relies on vestibular (otolith) processing.

Comment 9 What's the reason that you chose 1 cm inferior to the inion and 3 cm lateral to the right to stimulate the cerebellar hemisphere? Does use of these anatomical landmarks mean that there was no use of neuronavigation or individual brain MRIs to apply TMS at specific target regions in this study? Response:

The coil position was selected on the basis of previous studies targeting the cerebellar hemisphere (e.g., Matsugi et al., 2024; Hardwick et al., 2014). In addition, group-level EF simulations confirmed that this coil position induced strong EFs in the right hemisphere, which was not the case for the vermis (please see Extended Data Figure 1-4 in the revised text). Neuronavigation and individual MRI-based targeting were not used in this study, which is now clearly stated in the Materials and Methods and Limitations sections of the revised manuscript.

Changes:

Materials and Methods The neuronavigation system and individual head magnetic resonance imaging (MRI) were not used to target the brain regions. Instead, we simulated the electric fields (EFs) induced by TMS using high-resolution generic head models at the group level, with the anatomical landmark (inion) serving as the reference point for coil placement. Coil placement was determined on the basis of the simulation results (see below).

Limitations Second, we did not employ individual MRI-based neuronavigation to guide coil placement. Instead, coil positions were determined using an anatomical landmarks (the inion), informed by EF simulations in a set of high-resolution generic head models. Although the vermis seems to be less morphologically variable across individuals compared with other brain regions, such as the motor cortex (Eichert et al., 2016; Steel et al., 2017), this approach may still have introduced variability in effective targeting, particularly given the depth of the cerebellar vermis. However, it is also worth noting that even when neuronavigation systems are employed, non-negligible localization errors have been reported (Nieminen et al., 2022).

Comment 10 Will the TMS of double cone coil reach comparable depth on the three stimulation locations? Response:

We thank the reviewer for this thoughtful question regarding whether the double-cone coil used in our study could achieve comparable depth across the three stimulation sites (cerebellar vermis, cerebellar hemisphere, and visual cortex). As noted, anatomical differences among these sites can lead to variation in stimulation depth and focality.

To address this concern more directly, we calculated and visualized the distribution of the induced EF on sagittal slices. The analyses and figures illustrate that the cerebellar vermis is located in a deep midline region, and thus the EF induced at the surface of the vermis is attenuated compared with the other regions.

Despite this finding, we observed significant modulation of visual vertical bias only when targeting the vermis, with no comparable effect for the cerebellar hemisphere or visual cortex. This result suggests that the cerebellar vermis may have a functionally specific role in visuo-vestibular integration, independent of the physical ease or difficulty of stimulation. We have added new figures showing the EF distribution in the sagittal plane to the text (please see Extended Data Figure 1-2).

Changes:

Figure 1 Extended Data Figure 1-2 shows sagittal slices of the EF distribution in the vermis and V1-2 conditions. Extended Data Figure 1-3 represents the depth-dependent changes in EF from the coil surface in the vermis condition. See Extended Data Figures 1-1 and 1-4 for the EF distribution over the ventral (caudal) region of the cerebellum in the vermis condition in Experiment 1 and the EF distribution in the hemisphere condition (1 cm below and 3 cm to the right of the inion) in Experiment 2, respectively.

Extended Data Figure 1-2 Sagittal slice illustrating the EF distribution (V/m) in the vermis and V1-2 conditions. Each black dot represents the location of the maximum EF within each ROI. EF, electric field; ROI, region of interest.

Comment 11 Did not find JND in the paper (Barnett Cowan et al., 2010) you cited. If your fitted psychometric function is symmetric, (*LA*75−*SVV*) should be same as (*SVV*− *LA*25). The equation for JND can be simplified as (*LA*75−*SVV*). You can call this slope, but not appropriate as JND.

Response:

Thank you for your careful reading and for pointing out the issue regarding the JND. As the reviewer noted, Barnett-Cowan et al. (2010) did not explicitly describe the calculation of the JND, although they evaluated precision in the SVV task on the basis of the psychometric curve. Thus, citing their paper in this context was inappropriate, and we have removed the reference accordingly.

Additionally, we fitted a symmetric psychometric function, allowing us to calculate the JND as the difference in stimulus level between LA75 and the point of subjective equality (PSE), i.e., SVV. Indeed, previous studies have defined the JND in this way when using symmetric functions (e.g., Yao et al., 2019, J Neurosci; Rösler et al., 2015, J Neurophysiol), and have used this measure to assess judgment precision. We have revised the manuscript to clarify this point and added some citations.

Changes:

Materials and Methods We first calculated the stimulus values (i.e., line angles) at a response rate of 75% (〖LA〗_75) on the basis of the fitted psychometric function. Because the fitted function was symmetric, the JND for each phase and rTMS condition was determined as the difference between the line angle at a response rate of 50% (i.e., SVV) and the 〖LA〗_75 (Gescheider, 1997; Yao et al., 2019; Vatakis et al. 2018; Rösler et al., 2015). In this symmetric case, the JND can also be considered as an index of the slope of the psychometric function around the SVV point, with larger JNDs indicating less precise perceptual judgments.

Comment 12 Did you calculate the effect of OKS on SVV precision or just compare precision in the No-OKS condition pre- and post-TMS? Response:

We appreciate this question.

We calculated the OKS effect on the SVV but not on SVV precision (JND). In response to your question, we have quantified the OKS effect on the JND for each phase and rTMS condition, termed OKS-effectJND, by subtracting the JND in the No-OKS condition from that in the OKS condition. OKS-effectJND was positive regardless of phase and rTMS condition (mean {plus minus} standard deviation; Vermis, 1.90 {plus minus} 1.24{degree sign} and 1.43 {plus minus} 0.65{degree sign}; V1-2, 2.51 {plus minus} 1.54{degree sign} and 2.09 {plus minus} 2.57{degree sign}; sham, 1.95 {plus minus} 0.95{degree sign} and 1.01 {plus minus} 0.67{degree sign} in the pre- and post-rTMS phases, respectively). These results indicate that the OKS led to imprecision in visual vertical judgments, consistent with previous studies (Dockheer et al., 2018; Ward et al. 2017). We added these results to the text.

We also did not compare the JND in the No-OKS condition between the pre- and post-rTMS phases. Thus, we additionally compared the JND in the No-OKS condition between phases using another LMEM, with Vermis, V1-2, Phase, and their interaction as fixed effects and Participant and Group as random intercepts. No significant fixed-effect factors were found (all at p > 0.47, η_p^2 {less than or equal to} 0.02), indicating that precision in visual vertical judgments in the absence of visual rotational cues was not affected by rTMS over the vermis and V1-2. We have also added these results to the text.

Changes:

Results JND Figure 3A shows the JND in the No-OKS condition for each rTMS condition and phase. The JNDs tended to slightly increase (i.e., decrease in precision) after rTMS exposure in all rTMS conditions. For the ΔJND, LMEM analysis revealed that the effects of Vermis (β = -0.33, t = -0.32, p = 0.74, η_p^2 = 0.00) and V1-2 (β = 0.47, t = 0.47; p = 0.64, η_p^2 = 0.00) were not significant (Fig. 3B). The negative coefficient of 〖JND〗_pre was significant (β = -0.70, t = -5.13; p < 0.001, η_p^2 = 0.32).

To further examine the effects of rTMS, we compared JNDs in the No-OKS condition between the pre- and post-rTMS phases using another LMEM analysis with Vermis, V1-2, Phase, and their interaction as fixed effects and Participant and Group as random intercepts. No significant fixed-effect factors were found (all at p > 0.47, η_p^2 {less than or equal to} 0.02). Together, these results suggest that precision in visual vertical judgments in the absence of visual rotational cues may not be affected by rTMS over the cerebellar vermis, in contrast to visual dependency.

To confirm whether the OKS influenced judgment precision, we quantified the OKS-effect on the JND (termed OKS-effectJND) by subtracting the JND in the No-OKS condition from that in the OKS condition. OKS-effectJND was positive regardless of phase and rTMS condition (mean {plus minus} standard deviation: vermis, 1.90 {plus minus} 1.24{degree sign} and 1.43 {plus minus} 0.65{degree sign}; V1-2, 2.51 {plus minus} 1.54{degree sign} and 2.09 {plus minus} 2.57{degree sign}; sham, 1.95 {plus minus} 0.95{degree sign} and 1.01 {plus minus} 0.67{degree sign} in the pre- and post-rTMS phase, respectively). These results indicate that visual motion cues reduced the precision of visual vertical judgments, consistent with previous findings (e.g., Dockheer et al., 2018; Ward et al., 2017 [trend only]).

Comment 13 Results Suggest moving the first paragraph to Methods.

Response:

As the reviewer suggested, we have moved the description regarding the inclusion/exclusion of the data in Experiments 1 and 2 to the Materials and Methods section.

Changes:

Materials and Methods (Statistical analysis) In Experiment 1, data from two participants were excluded from the group analysis because of significant data loss (approximately 1/3 of all trials; ID: 9) and non-completion of the experiment because of nausea (although without seizures or syncope) during OKS exposure (ID: 13). After excluding trials with no response within the time limit (2 s), 99.3% of the total trials were included in the group analysis. In Experiment 2, no participants were excluded. After removing trials without responses, 99.3% of the total trials were retained for the group analysis.

Comment 14 Line 312, please keep Sub ID number here consistent with the figure 2A legend.

Response:

We apologize for the incorrect description in the legend of Fig. 2A; the ID was 3. We have corrected this error.

Changes:

Figure 2 (A) SVV task performance in each OKS condition of the sham condition for a representative participant (Sub ID = 3, CW group).

Comment 15 Line 314 and line 317, make sure the numbers match the figure.

Response:

We apologize for this mistake, which has been corrected (participant ID = 3).

Comment 16 In Figure 2A, in pre-rTMS plot OKS condition, the proportion of CW response of one circle is about 0.25 but its neighbor circles are zero. So this could be caused by a couple of trials during which the participant pressed the wrong button. In post-rTMS plot, the line angle goes beyond the range you tested.

Response:

Thank you for your careful observations. Regarding the proportion of CW responses in the pre-rTMS phase and OKS condition, we have double-checked the raw data and confirmed that there were no recording errors or button-press mistakes. The observed proportion of 0.25 at a line angle of -2.5{degree sign}, with the neighboring angles showing zero, is consistent with the participant's psychophysical responses at those specific angles under the OKS condition, and seems to represent the nature of perceptual decision-making variability rather than an input error.

Regarding the post-rTMS plot, we found that a circle marker had been mistakenly plotted at an incorrect line angle position. This error has been corrected in the revised figure.

Comment 17 Can you do a separate correlation analysis between ΔOKS-effect and OKS-effectpre for three rTMS conditions? Any significant differences in OKS-effectpre among three rTMS conditions? Response:

Thank you for your suggestion.

Following this comment, we conducted separate correlation analyses between ΔOKS-effect and OKS-effectpre for each rTMS condition. A significant correlation was found in the vermis condition (r = -0.68, p =0.001) but not in the V1-2 (r = 0.12, p = 0.61) or sham condition (r = -0.25, p = 0.27). As the OKS-effectpre was already controlled for as a covariate in the linear mixed-effects model (LMEM) used in our primary analysis, we think that including these additional results in the main manuscript would not substantially contribute to our conclusions. Therefore, we have chosen not to include these additional analyses, but we would be happy to report these results in detail upon request.

In addition, we newly compared the OKS-effectpre values (vermis, 5.66 {plus minus} 4.66{degree sign}; V1-2, 4.92 {plus minus} 5.31{degree sign}; sham, 5.43 {plus minus} 5.10{degree sign}) across the three rTMS conditions using another LMEM (with two separate fixed-effect factors of Vermis and V1-2, and two random-effect factors of Participant and Group). We found no significant effect of Vermis (β = 0.22, t = 0.41, p = 0.69) or V1-2 (β = 0.20, t = 0.36, p = 0.72), indicating no significant difference in OKS-effectpre between the rTMS conditions. We have added this additional result to the text.

Changes:

Results Additionally, we checked whether the OKS-effect in the pre-rTMS condition (i.e., OKS-effectpre) differed across rTMS conditions using another LMEM with two separate fixed-effect factors (Vermis and V1-2) and a random-effect factor (participant). No significant effects of Vermis (β = 0.22, t = 0.41, p = 0.69, η_p^2 = 0.00) or V1-2 (β = 0.20, t = 0.36, p = 0.72, η_p^2 = 0.00) were observed, indicating no significant difference in the OKS-effectpre between the rTMS conditions.

Comment 18 Line 326-327, this sentence is confusing. Please correct.

Response:

We have revised this sentence.

Changes:

Results The LMEM revealed a significant negative effect of Vermis on ΔOKS-effect (β = -1.39, t = -0.10, p = 0.33, η_p^2 = 0.03), whereas the effect of V1-2 was not significant (β = -0.56, t = -0.10, p = 0.33, η_p^2 = 0.03). There was a significant negative coefficient for the OKS-effectpre (β = -0.40, t = -3.82, p < 0.001, η_p^2 = 0.28; Fig. 2D), implying that participants with a larger OKS-effect in the pre-rTMS phase showed a smaller ΔOKS-effect. These results indicate that visual dependency in the SVV judgment task was attenuated by 1-Hz rTMS over the cerebellar vermis.

Comment 19 What's the effect of OKS on SVV precision? Response:

As described in our response to Comment 12, we additionally examined the effect of OKS on SVV precision by comparing JNDs between the OKS and No-OKS conditions. The observed positive value of OKS-effect indicated that, consistent with previous studies (Dockheer et al., 2018; Ward et al., 2017), the addition of OKS (i.e., visual motion cues) reduced the precision of visual vertical judgments, as noted in the revised Results section.

Comment 20 Line 347-348, please clarify the coefficient of JND Pre Response:

The coefficient of JND Pre (β = -0.70) has been added to the text. Additionally, we have added the β value of each variable for each LMEM to the text.

Changes:

The negative coefficient of 〖JND〗_pre was significant (β = -0.70, t = -5.13; p < 0.001, η_p^2 = 0.32).

- # Reviewer 2 Comment 1 Overall, this experimental study sheds further light on the cerebellum's role in modulating the multisensory integration of visual and vestibular input for spatial orientation. Strengths of the study are the hypothesis-driven approach with a sound rationale for performing this study and the well-described methods. But also the statistical analysis and the interpretation of the results are straight-forward and reasonable. Thus, I do not see any major issues. However, a few aspects require the authors' attention. This includes both the lack of neuro-navigation based on individual MR-imaging to identify the target locations and a discussion why rTMS was preferred over theta-burst stimulation.

Response:

We thank the reviewer for the positive and constructive comments. We have addressed the points raised regarding the use of neuronavigation and the choice of rTMS in the revised manuscript (please see below for details).

Comment 2 Abstract:

Line 7: please provide a more detailed location. cerebellar vermis? hemispheres?? Response:

We have revised this description as shown below.

Changes:

Abstract To address this gap, we conducted two experiments with 42 healthy participants to evaluate the impact of repetitive 1-Hz transcranial magnetic stimulation (rTMS) over the posterior cerebellar vermis on visual dependency, quantified by the subjective visual vertical bias induced by rotating optokinetic stimulation (OKS).

Comment 3 Material and methods:

Line 99: please describe with more detail how the coil was placed in the sham condition. How remote was the coil from the surface of the skin or did it touch the skin but just had a perpendicular angle? Response:

One edge of the coil touched the scalp to reproduce the auditory click and somatosensory sensation without effective brain stimulation. We have described this in the text.

Changes:

Materials and Methods In the sham condition, the coil was set at the same position as in the vermis condition, but was tilted backward at a 90{degree sign} angle relative to the scalp surface (i.e., the handle was oriented backward), with one edge touching the scalp to reproduce the auditory click and somatosensory sensation without effective brain stimulation.

Comment 4 Line 140: the SVV paradigm used is often referred to an „alternate forced choice paradigm". It might be worthwhile referring to this for clarity.

Response:

Thank you for the suggestion. We have added "two-alternative forced-choice paradigm" to the text.

Changes:

Materials and Methods Then, a gray fixation point appeared in the center for 2 s ("Judgment" in Fig. 1A, lower panel), within which the participant judged whether the line orientation was tilted in the CW or CCW direction with respect to the gravitational vertical using a two-alternative forced-choice paradigm by pressing one of two keys on a customized numeric keypad (TK-TCM011SV; Elecom, Osaka, Japan) with the right and left thumbs.

Comment 5 Line 215: would it be possible to provide a sagital view in Fig 1C to illustrate the EF in the vermis. The reason is that the distance from the skin to the vermis can be several cm. Furthermore, a more technical description of the capabilities of the TMS coil used would be nice. Especially regarding ist capability to reach deeper brain areas.

Response:

We thank the reviewer for this important suggestion.

To address your comment, we have added a sagittal slice showing the EF distribution for the vermis condition (Extended Data Figure 1-2) to illustrate the depth and extent of stimulation. Although the cerebellar vermis is relatively deep compared with more superficial cortical areas, this sagittal slice clearly illustrates that the EF induced at the vermis still reaches an adequate intensity (approximately 50 V/m).

In addition, we have now included a brief technical description of the stimulation coil in the revised Materials and Methods section. The double-cone coil (D-B80; MagVenture) used in this study is designed to achieve greater depth penetration than conventional figure-of-eight coils. Our EF simulations confirmed this: for the same coil orientation and input current, the maximum EF at the cerebellar midline (vermis) was approximately 68.3 V/m using the double-cone coil, whereas the EF with a figure-of-eight coil under comparable positioning would drop substantially below this value in deeper tissues because of its more focal but shallower field distribution (Laakso et al., 2015; Gomez-tames et al., 2020).

Changes:

Figure 1 Extended Data Figure 1-2 shows sagittal slices of the EF distribution in the vermis and V1-2 conditions. Extended Data Figure 1-3 represents the depth-dependent changes in EF from the coil surface in the vermis condition. See Extended Data Figures 1-1 and 1-4 for the EF distribution over the ventral (caudal) region of the cerebellum in the vermis condition in Experiment 1 and the EF distribution in the hemisphere condition (1 cm below and 3 cm to the right of the inion) in Experiment 2, respectively.

Extended Data Figure 1-2 Sagittal slice illustrating the EF distribution (V/m) in the vermis and V1-2 conditions. Each black dot represents the location of the maximum EF within each ROI. EF, electric field; ROI, region of interest.

Materials and Methods The double-cone coil is designed for deeper brain stimulation, providing a broader magnetic field distribution that facilitates penetration into deep midline structures (Gomez-Tames et al., 2020) including the cerebellar vermis.

Comment 6 Line 227: please provide information about estimated depth of penetration, currently only EF distribution on the cerebellar surface is shown.

Response:

Thank you for this suggestion. We newly simulated the EF distribution for the vermis condition according to the depth from the coil surface, which apparently corresponds to the skin. The mean {plus minus} standard deviation penetration depth, defined as the depth at which the EF strength decreases to half of its value at the coil surface, was 19.7 {plus minus} 10.6 mm. This simulation result has been included in the revised manuscript (see Extended Data Figure 1-3 and its caption).

Changes:

Figure 1 Extended Data Figure 1-3 represents the depth-dependent changes in EF from the coil surface in the vermis condition.

Extended Data Figure 1-3 Depth-dependent changes in EF strength under the vermis condition. The horizontal axis indicates the depth (in mm) from the coil surface, which approximately corresponds to the scalp. Gray lines represent the EF strength in individual head models, whereas the black line shows the mean EF across the 18 models. The mean {plus minus} standard deviation penetration depth, defined as the depth at which the EF strength decreases to half of its value at the coil surface, was 19.7 {plus minus} 10.6 mm. EF, electric field.

Comment 7 Results Line 308: please briefly provide information about side effects of TMS (not only for those that dropped out). Did you observe any neck (muscle) pain, headaches etc...? Response:

We have added text detailing the absence of side effects of rTMS.

Changes:

Results No participants in either Experiments 1 or Experiment 2 showed any TMS-induced side effects such as neck muscle pain, headaches, or other discomfort.

Comment 8 Discussion:

In a limitations section the lack of individual neuro-navigation used to identify the target stimulation areas should be mentioned and potential downsides of using anatomical landmarks as the inion only should be discussed.

Response:

As suggested, we have added a dedicated "Limitations" section to the revised manuscript, which includes a discussion of the absence of neuronavigation and the use of generic anatomical models and anatomical landmarks (e.g., inion) for coil positioning. Although this approach enables group-level standardization, we acknowledge that it may introduce spatial variability in individual EF targeting. However, we also note that the cerebellar vermis is less affected by morphological interindividual differences than cortical structures, supporting the generalizability of our approach.

Changes:

Limitations Second, we did not employ individual MRI-based neuronavigation to guide coil placement. Instead, coil positions were determined using an anatomical landmarks (the inion), informed by EF simulations in a set of high-resolution generic head models. Although the vermis seems to be less morphologically variable across individuals compared with other brain regions, such as the motor cortex (Eichert et al., 2016; Steel et al., 2017), this approach may still have introduced variability in effective targeting, particularly given the depth of the cerebellar vermis. However, it is also worth noting that even when neuronavigation systems are employed, non-negligible localization errors have been reported (Nieminen et al., 2022).

Comment 9 Probably the authors could also briefly discuss why they preferred 1Hz rTMS over continous theta-burst stimulation (cTBS)? Response:

We appreciate this question. As the reviewer indicated, the cTBS is also an inhibitory rTMS technique, but 1-Hz rTMS seems to be safer (Rossi et al., 2009, 2021; Cole et al., 2023). We have provided a reason for the application of 1-Hz rTMS in this study in the Materials and Methods section.

Changes:

Materials and Methos We selected 1-Hz rTMS over continuous theta-burst stimulation; although they can produce similar levels of inhibition (Zafar et al., 2008), 1 Hz rTMS has a well-established safety profile and a lower risk of side effects (Rossi et al., 2009, 2021; Cole et al., 2023).

Comment 10 Figures Figure 1: there is a typo in Fig 1A: task familiarzation instead of familiarization Response:

We apologize for the typo, which has been corrected.

- #Editor Comment 1 # Synthesis After careful evaluation by two experts in the field, we find that your study addresses an important question regarding the cerebellum's contribution to visuo-vestibular integration. Both reviewers appreciated the hypothesis-driven design and methodological rigor, including the use of multiple control sites. However, several important concerns need to be addressed, and I encourage you to carefully consider and respond to each of these points in your revision.

Response:

We thank the Editor for the thoughtful summary and for the opportunity to revise our manuscript. We are pleased that both reviewers recognized the importance of our research question and the strengths of our experimental design, including the use of multiple control sites. In the revised manuscript, we have addressed all of the reviewers' comments carefully. We hope that the revisions adequately resolve the issues raised and improve the overall clarity and impact of our work.

Comment 2 Both reviewers highlighted limitations related to the strength of the statistical evidence and the use of generic head models. I suggest following Reviewer 2's recommendation to add a "Limitations" section to the Discussion. This section should address, among others, the following points:

- A justification of the sample size, particularly in light of the small and variable effects observed;

- A clarification regarding the use of generic head models instead of individual MRIs, and the absence of neuronavigation, including a discussion of the potential limitations this entails.

Response:

In accordance with Reviewer 2's recommendation, we have added a new "Limitations" section to the revised text. In this section, we now acknowledge the modest and variable effects observed in the data, and clarify our use of generic head models instead of individual MRI images and a neuronavigation system. We believe that these additions improve the transparency and interpretability of our findings.
