# Supplementary figures and images for: Cerebellum Involvement in Visuo-vestibular Interaction for the Perception of Gravitational Direction: A Repetitive Transcranial Magnetic Stimulation Study
Source: eNeuro. 2025 Jul 30;12(7):ENEURO.0111-25.2025. doi: 10.1523/ENEURO.0111-25.2025 (PMC12320761; doi:10.1523/ENEURO.0111-25.2025)

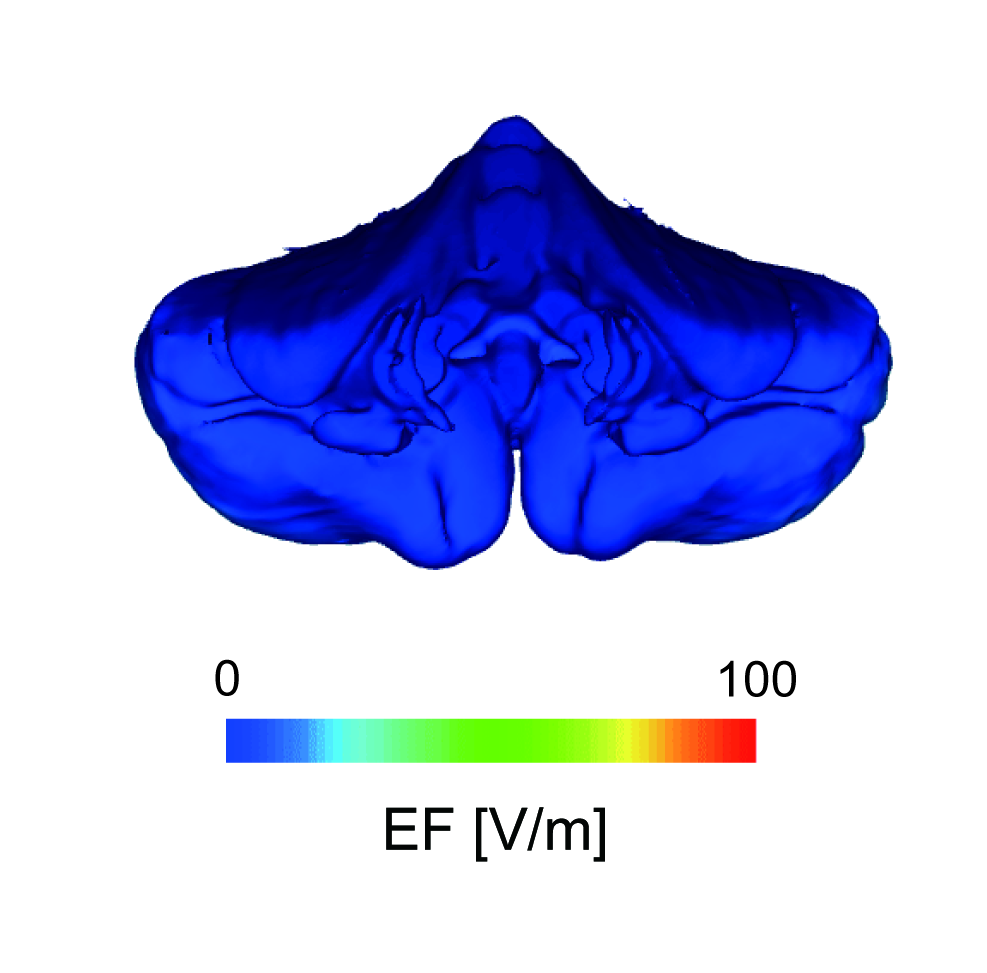

Supplement: Figure 1-3 — Estimated EF distribution (V/m) in the ventral region of the cerebellum under the vermis condition. The maximum and mean EF values within the ROI covering both flocculi were 24.1 V/m and 10.0 V/m, respectively. EF, electric field; ROI, region of interest. Download Figure 1-3, TIF file. [file eneuro-12-ENEURO.0111-25.2025-s004.tif]

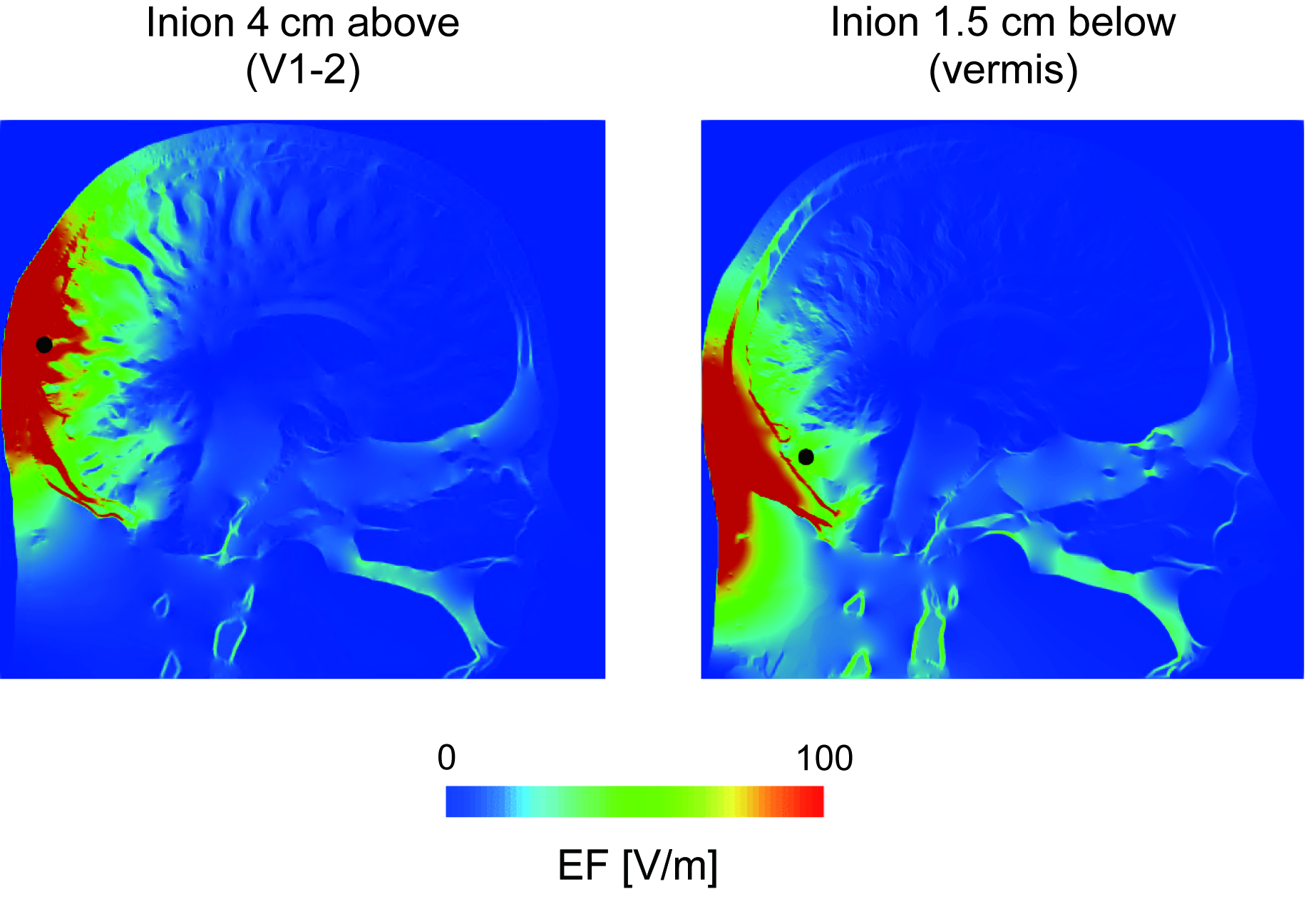

Supplement: Figure 1-4 — Sagittal slice illustrating the EF distribution (V/m) in the vermis and V1–2 conditions. Each black dot represents the location of the maximum EF within each ROI. EF, electric field; ROI, region of interest. Download Figure 1-4, TIF file. [file eneuro-12-ENEURO.0111-25.2025-s005.tif]

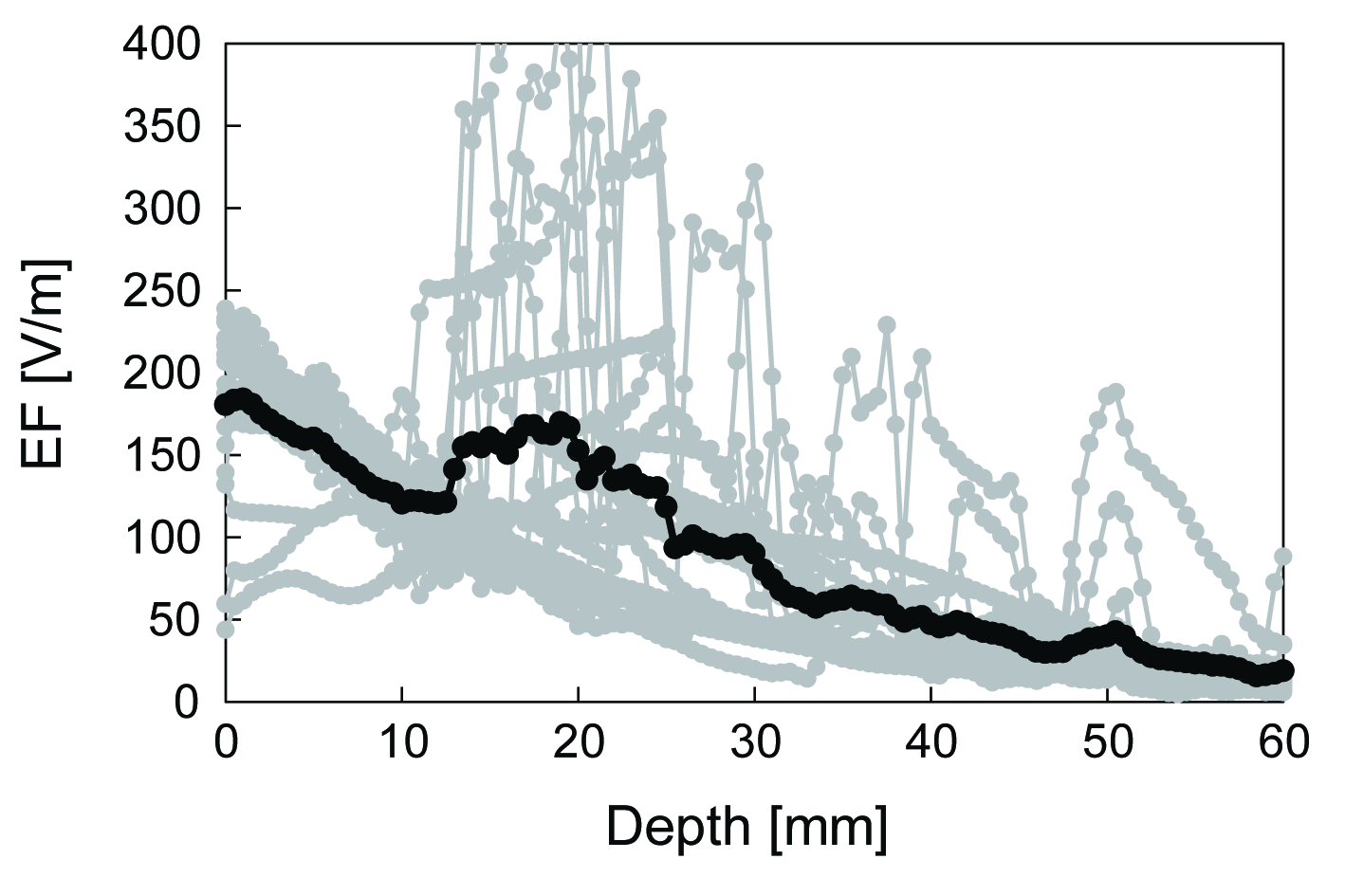

Supplement: Figure 1-5 — Depth-dependent changes in EF strength under the vermis condition. The horizontal axis indicates the depth (in mm) from the coil surface, which approximately corresponds to the scalp. Gray lines represent the EF strength in individual head models, whereas the black line shows the mean EF across the 18 models. The mean ± standard deviation penetration depth, defined as the depth at which the EF strength decreases to half of its value at the coil surface, was 19.7 ± 10.6 mm. EF, electric field. Download Figure 1-5, TIF file. [file eneuro-12-ENEURO.0111-25.2025-s006.tif]

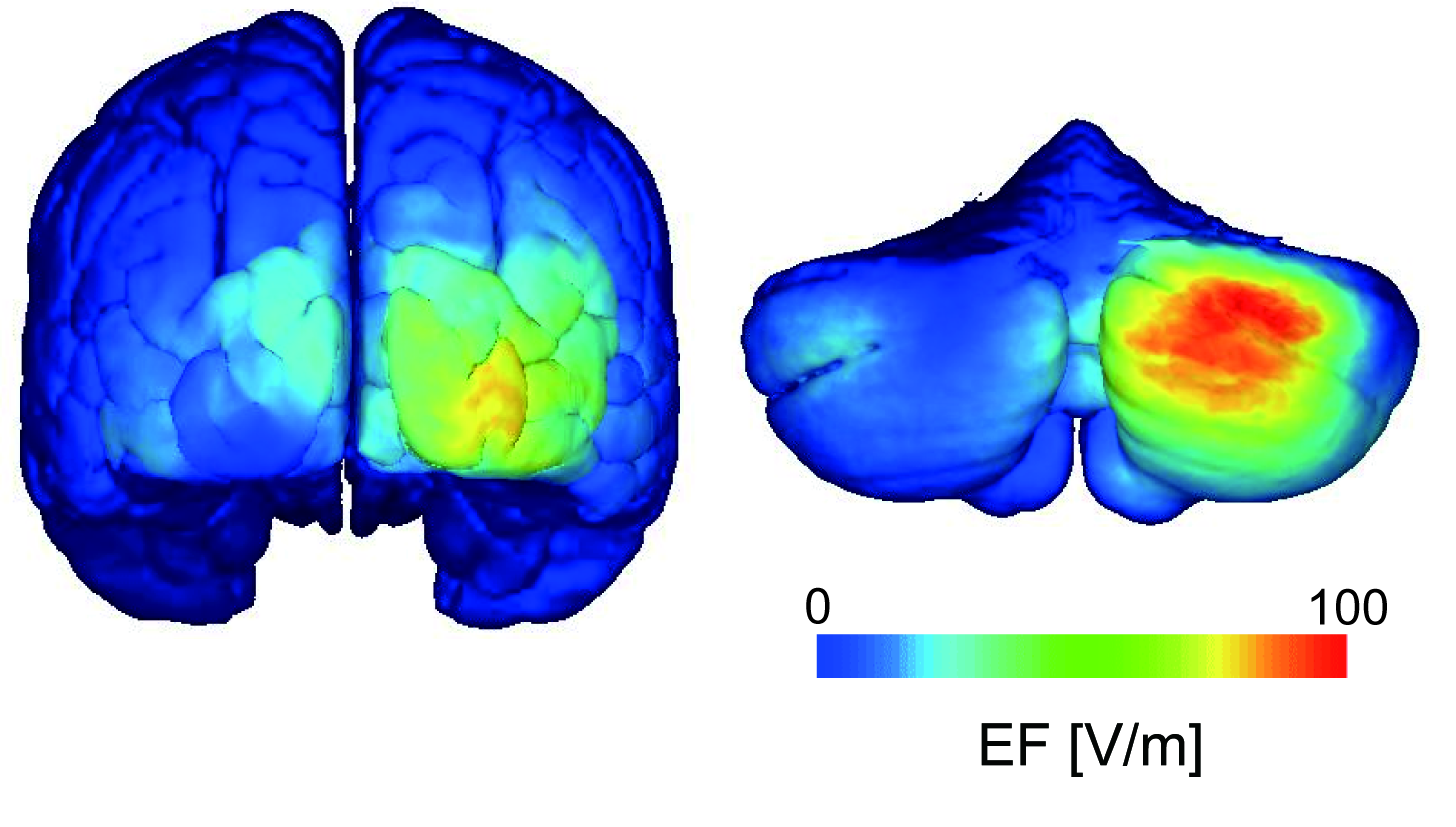

Supplement: Figure 1-6 — The estimated EF distribution (V/m) for the coil placement (1cm below and right to the inion) assigned to the hemisphere condition (Experiment 2). EF, electric field. Download Figure 1-6, TIF file. [file eneuro-12-ENEURO.0111-25.2025-s007.tif]
